# Characterization and Genome Analysis of a Novel Vibrio Alginolyticus Phage and Its Biocontrol Efficacy in Pearl Oyster Aquaculture

**DOI:** 10.3390/ijms27209385

**Published:** 2026-10-21

**Authors:** Xuyang Chen, Xuejing Li, Yiyang Zhu, Yantao Liang, Xing Ren, Shumeng Zhang, Junyi Tao, Kailiang Zou, Longfei Lu

**Affiliations:** 1 Guangxi Laboratory of Oceanography, Fourth Institute of Oceanography, Ministry of Natural Resources, Beihai 536000, China; chenxuyang@4io.org.cn (X.C.); lixuejing@4io.org.cn (X.L.); renxing@4io.org.cn (X.R.); zhangshumeng@4io.org.cn (S.Z.); 2 Key Laboratory of Tropical Marine Ecosystem and Bioresource, Fourth Institute of Oceanography, Ministry of Natural Resources, Beihai 536000, China; 3 Laboratory of Viral & Microbial Diversity, Ocean University of China, Qingdao 266003, China; zhuyiyang@stu.ouc.edu.cn (Y.Z.); liangyantao@ouc.edu.cn (Y.L.); 4 College of Oceanography and Ecological Science, Shanghai Ocean University, Shanghai 201306, China; 2315110@st.shou.edu.cn; 5 College of Oceanography, Beibu Gulf University, Qinzhou 535011, China; 19128077762@163.com

**Keywords:** *Vibrio alginolyticus*, *Vibrio* phage, morphology and genomic characterization, taxonomic assignment, aquaculture biocontrol

## Abstract

A novel lytic bacteriophage, designated vB_ValS-L9, infecting the potentially pathogenic bacterium *Vibrio alginolyticus* L4, was isolated from a coastal aquaculture area in Beihai, China. Transmission electron microscopy revealed that vB_ValS-L9 possesses an icosahedral head (56.50 ± 4.19 nm in diameter) and a long tail (143.03 ± 5.32 nm in length), exhibiting a typical siphovirus-like morphology within the class *Caudoviricetes*. The phage demonstrated stability across various temperatures, pH levels, and salinities. Genomic sequencing showed that vB_ValS-L9 has a circular double-stranded DNA genome of 44,025 bp with a G + C content of 43.56% and 61 predicted ORFs. No tRNA, virulence, or antibiotic resistance genes were identified. Genomic and phylogenetic analyses, including ANI, VICTOR, VIRIDIC, and conserved protein phylogenies, identified vB_ValS-L9 as a novel species that, together with *Vibrio* phages Artemius and VapS30, forms a new genus in the class *Caudoviricetes*, for which the name *Artemiovirus* is proposed. Furthermore, in vivo trials using juvenile pearl oysters (*Pinctada martensii*) revealed that vB_ValS-L9 provides substantial prophylactic and therapeutic protection against *V. alginolyticus* L4 infection, especially when the phage was administered before bacterial challenge. Collectively, these findings suggest that vB_ValS-L9 shows potential as a safe, environmentally robust, and promising biocontrol candidate for managing vibriosis in aquaculture.

## 1. Introduction

The genus *Vibrio* encompasses a diverse group of Gram-negative, halophilic bacteria that are ubiquitous in marine and estuarine ecosystems [1,2]. Several *Vibrio* species, including *Vibrio parahaemolyticus*, *Vibrio alginolyticus*, *Vibrio anguillarum*, and *Vibrio harveyi*, are major pathogens in mariculture and are responsible for severe disease outbreaks that cause substantial economic losses globally [3,4,5,6]. Infections such as vibriosis can result in mass mortality of farmed fish, crustaceans, and mollusks, threatening the sustainability and profitability of the aquaculture industry [7,8]. Furthermore, certain pathogenic *Vibrio* species are also zoonotic human pathogens capable of causing foodborne or direct-contact infections [9,10,11]. Among them, *V. alginolyticus* is one of the most frequently encountered species in warm coastal waters [12]. Initially isolated from seawater and characterized for its pronounced alginate-degrading ability [13], *V. alginolyticus* was later recognized as an important opportunistic pathogen for both marine animals and humans [14]. In aquaculture settings, it has been implicated in acute vibriosis outbreaks affecting a wide range of economically important species, including oyster, crab, abalone, fish, and shrimp [15,16,17,18,19,20].

Compounding this threat, the overuse and misuse of conventional antibiotics have accelerated the emergence of multidrug-resistant strains, complicating infection management and increasing mortality rates in both aquaculture animals and humans [21]. Multidrug-resistant “superbugs” are currently recognized among the top threats to global health [22]. Therefore, it is imperative to explore alternative, eco-friendly biocontrol strategies, such as phage therapy.

Bacteriophages (phages for short), the natural viral predators of bacteria, have gained widespread attention as sustainable alternatives to antibiotics for controlling pathogenic *Vibrio* in aquaculture [6,23,24,25]. To date, numerous *Vibrio* phages have been isolated from diverse marine environments, revealing remarkable morphological and genomic diversity. The vast majority of these phages are tailed viruses classified within the class *Caudoviricetes* (NCBI GenBank, accessed on 15 May 2026). Despite this recognized diversity, the number of isolated and sequenced *Vibrio* phages remains limited relative to the immense viral richness predicted by metagenomic surveys. Moreover, phylogenetic analyses based on whole-genome nucleotide sequences (e.g., ViPTree, VIRIDIC) frequently reveal that many newly isolated *Vibrio* phages cannot be reliably assigned to any recognized family or genus, highlighting significant taxonomic gaps [26,27,28,29]. Under the latest ICTV taxonomy releases (ratified in 2025), a considerable proportion of phage genome sequences deposited in public databases remain unassigned at multiple taxonomic ranks [30], impeding our comprehensive understanding of phage evolution and diversification. Consequently, the isolation and in-depth characterization of novel lytic *Vibrio* phages are needed not only to bridge these taxonomic and ecological knowledge gaps but also to expand the candidate phage pool for targeted biocontrol applications.

In the present study, a novel lytic *V. alginolyticus* phage, designated vB_ValS-L9, was isolated from coastal aquaculture seawater. Its morphological features, physicochemical stability, and genomic properties were systematically characterized, and its taxonomic assignment was delineated through comprehensive comparative genomic and phylogenetic analyses. Furthermore, its in vivo prophylactic and therapeutic efficacy was evaluated using a juvenile pearl oyster (*Pinctada martensii*) challenge model, and the effect of varying the timing of phage administration on therapeutic efficacy was investigated. This study broadens the current understanding of *V. alginolyticus* phage biology and introduces a safe, highly effective candidate for phage-based biocontrol in aquaculture.

## 2. Results

### 2.1. Phage Isolation, Morphology, Host Range, and Infection Kinetics

#### 2.1.1. Phage Isolation and Morphological Characterization

Phage vB_ValS-L9 was isolated from surface seawater collected from an aquaculture area of Beihai, China, using *Vibrio alginolyticus* L4 as the indicator host. On double-layer agar plates, vB_ValS-L9 produced small, clear, and round plaques after 24 h of incubation at 28 °C, demonstrating robust lytic activity against the host strain. Chloroform sensitivity assays showed that viral infectivity remained essentially unchanged across all tested chloroform volume fractions (1%, 10%, and 50%, *v*/*v*), confirming that the vB_ValS-L9 virion is non-enveloped and lacks essential structural lipids.

**Figure 1:**
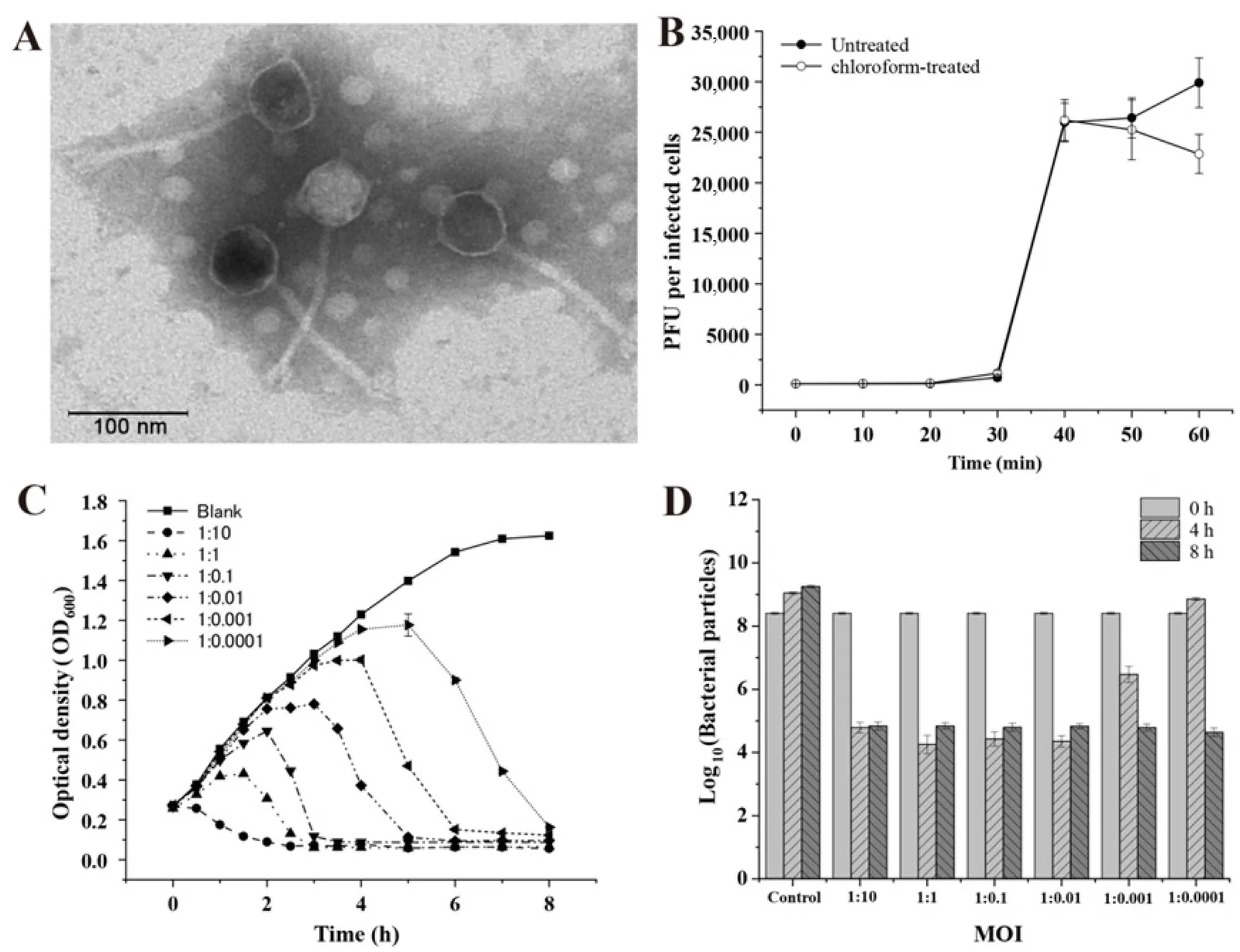
Morphological and infection dynamics of phage vB_ValS-L9. (**A**) Transmission electron microscopy image of *Vibrio* phage vB_ValS-L9. (**B**) One-step curve of phage vB_ValS-L9. (**C**) Killing curves of *Vibrio alginolyticus* L4 by phage vB_ValS-L9 infection at different MOIs. Error bars represent the standard deviations of triplicate samples. (**D**) Bacterial abundance at 4 h and 8 h at different MOIs. Results are presented as mean values ± SD and expressed on log_10_ scale.

Transmission electron microscopy (TEM) examination revealed that vB_ValS-L9 possesses an icosahedral head with a diameter of 56.50 ± 4.19 nm and a long, flexible, non-contractile tail measuring 143.03 ± 5.32 nm in length (Figure 1A). Based on these morphological characteristics, vB_ValS-L9 exhibits a typical siphovirus-like morphotype, classifying it within the class *Caudoviricetes* (traditionally classified under the former family *Siphoviridae*).

#### 2.1.2. Host Specificity and Infection Kinetics

The host range of phage vB_ValS-L9 was evaluated against 53 marine bacterial isolates, comprising 43 *Vibrio* strains, seven *Shewanella* strains, two *Pseudoalteromonas* strains, and one *Sulfitobacter* strain (Table S1). Spot assays revealed that vB_ValS-L9 formed distinct, clear plaques exclusively on its original isolation host, *V. alginolyticus* L4, whereas no lytic activity or plaque formation was observed across the remaining 52 tested strains.

To characterize the replication dynamics of phage vB_ValS-L9, a one-step growth curve assay was performed at a multiplicity of infection (MOI) of 0.01 using exponentially growing *V. alginolyticus* L4. The growth curve exhibited a latent period of approximately 40 min, followed by a rapid burst phase that yielded an estimated burst size of 231 PFU per infected cell (Figure 1B). To further assess its bactericidal potency under varying infection pressures, liquid lysis kinetics were monitored across MOIs spanning 0.001 to 10 (Figure 1C). Notably, even at the lowest tested MOI (0.001), phage vB_ValS-L9 exerted robust bactericidal activity against strain L4, as evidenced by a profound reduction in culture turbidity (OD_600_) compared to the uninfected control after both 4 h and 8 h of co-incubation (Figure 1D).

### 2.2. Environmental Stability of Phage Vb_vals-L9

**Figure 2:**
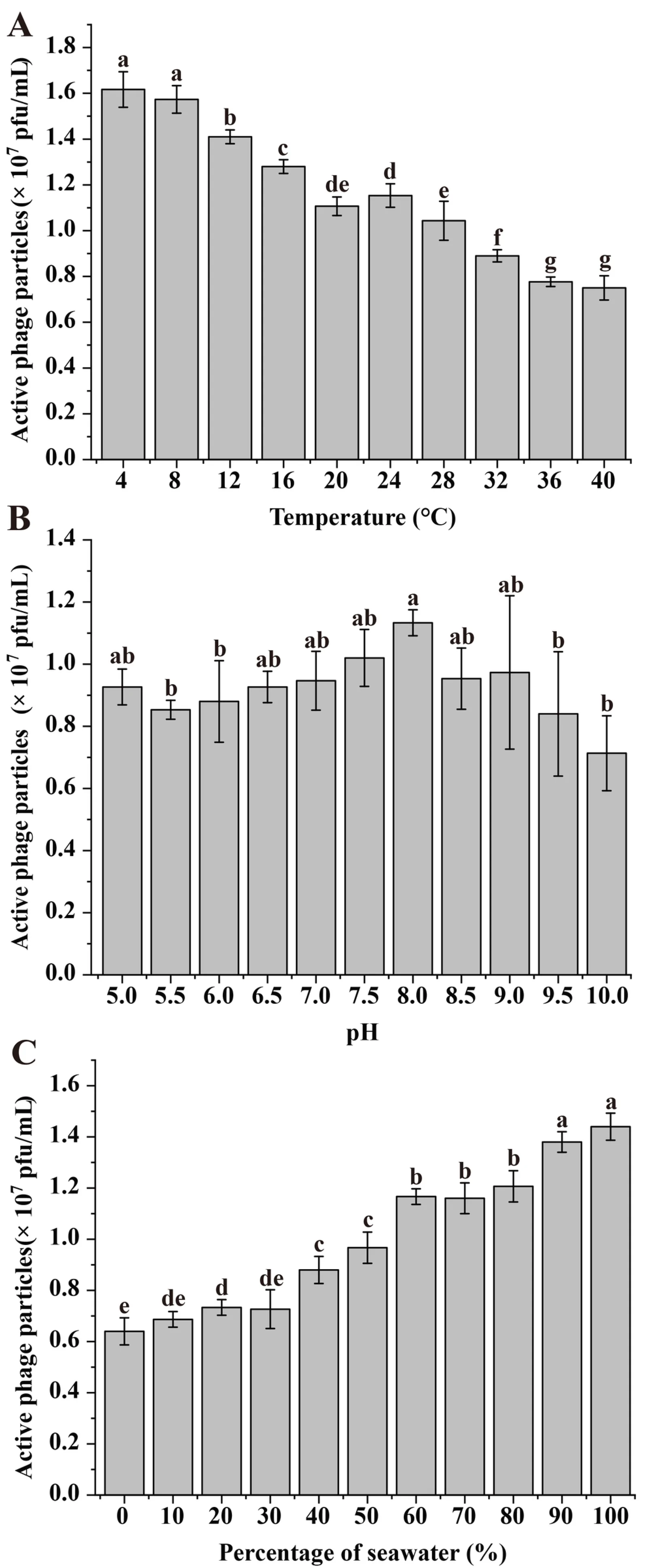
The stability of phage vB_ValS-L9 under different environmental conditions. (**A**) Stability of phage vB_ValS-L9 at different temperatures. (**B**) Stability of phage vB_ValS-L9 under different pH conditions. (**C**) Stability of phage vB_ValS-L9 under different salinity conditions. Error bars represent the standard deviations of triplicate samples. Statistically significant differences (*p* < 0.05) are indicated by different lowercase letters.

To evaluate the environmental resilience of phage vB_ValS-L9, its stability under various temperature, pH, and salinity conditions was systematically determined following a 24 h exposure period (Figure 2). Thermal stability assays indicated an inverse relationship between temperature and viral viability; nevertheless, phage vB_ValS-L9 exhibited moderate thermostability, retaining a 46.39% survival rate even after incubation at 40 °C (Figure 2A). In pH stability tests, vB_ValS-L9 demonstrated a notable tolerance across a broad pH spectrum ranging from 5.0 to 10.0, with optimal infectivity observed at physiological marine pH (pH 8.0) (Figure 2B). Notably, the phage retained approximately 81.76% and 62.94% of its initial titer under acidic (pH 5.0) and alkaline (pH 10.0) conditions, respectively. Regarding salinity tolerance, vB_ValS-L9 maintained substantial lytic capability across diverse salinity gradients (Figure 2C). Although viral titers displayed a slight, progressive decline when seawater concentration exceeded 60% (~20.40‰ salinity), the phage preserved more than 44.44% of its maximum viability even under pronounced hypotonic stress (10% seawater, ~3.40‰ salinity).

### 2.3. Genomic Features and Functional Annotation

#### 2.3.1. General Genomic Features and Functional Modularity

**Figure 3:**
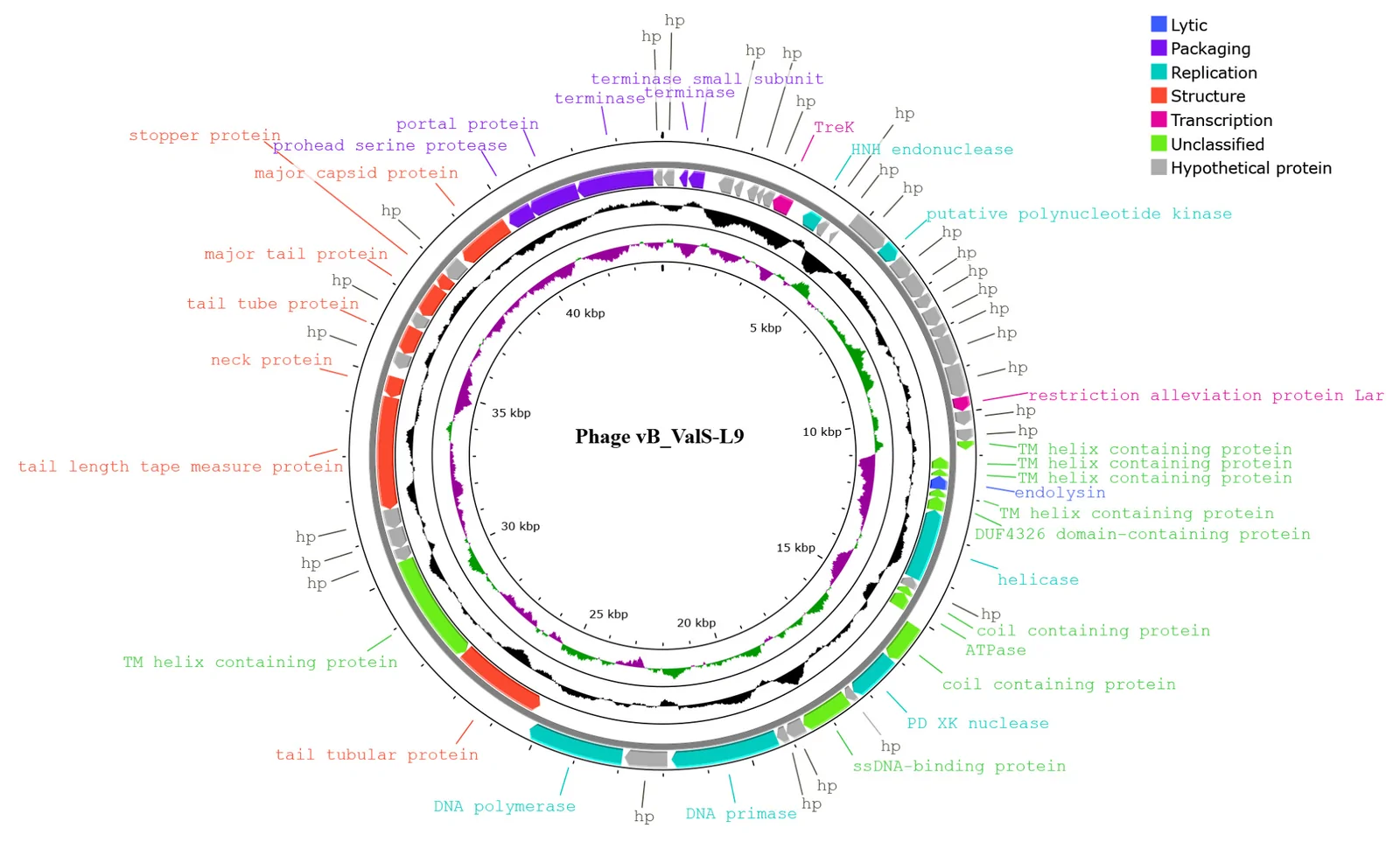
Circular genomic map of phage vB_ValS-L9. Predicted ORFs are color-coded according to their functional categories, as indicated in the legend. Arrows represent coding regions and indicate the direction of transcription. The abbreviation “hp” stands for hypothetical protein. From outside to inside: ORFs, GC content, GC skew, the scale.

High-throughput sequencing and de novo assembly yielded a complete, circular, double-stranded DNA genome for phage vB_ValS-L9, comprising 44,025 bp with an overall G + C content of 43.56%. Quality evaluation via CheckV corroborated that the assembled genome was 100% complete and circular, with no detectable host contamination. A total of 61 ORFs were predicted across the genome, yielding a high coding density of 95.28% (Figure 3; Table S2). Among these, 31 ORFs (50.82%) were assigned putative biological functions based on homology searches and conserved domain analysis, whereas the remaining 30 ORFs (49.18%) were designated as hypothetical proteins with uncharacterized functions. Comprehensive screening confirmed the absence of tRNA genes, antimicrobial resistance genes, virulence factor determinants, and lysogeny-associated marker enzymes (e.g., integrases, recombinases, and excisionases), reinforcing the strictly lytic lifestyle and biosafety of vB_ValS-L9.

The functionally annotated ORFs were organized into five distinct modules according to their predicted roles in the phage life cycle: DNA replication and repair (6 ORFs: ORF10, ORF14, ORF31, ORF36, ORF41, and ORF43), viral packaging and morphogenesis (5 ORFs: ORF2, ORF3, ORF58, ORF59, and ORF60), structural components (7 ORFs: ORF44, ORF49, ORF50, ORF52, ORF54, ORF55, and ORF57), host lysis (1 ORF: ORF28), and transcriptional regulation (2 ORFs: ORF9 and ORF22). The remaining 10 ORFs lacked clear functional assignments but were predicted to encode proteins containing conserved structural features, including transmembrane helices (ORF25, ORF26, ORF27, ORF29, and ORF45), coiled-coil domains (ORF33 and ORF35), an ATPase (ORF34), a single-stranded DNA-binding protein (ORF38), and a DUF4326 domain-containing protein (ORF30).

#### 2.3.2. Annotation of Predicted Orfs

ORF28 is annotated as an endolysin containing a conserved peptidoglycan hydrolase domain and may participate in bacterial cell wall degradation during progeny release [31]. Virion packaging and capsid maturation are orchestrated by five coordinated proteins: the terminase (ORF2 and ORF60) and terminase small subunit (TerS, ORF3) of the terminase complex mediate viral DNA recognition, cleavage, and translocation into the procapsid via the portal protein (ORF59) [32,33], while a prohead serine protease (ORF58) drives scaffolding protein cleavage and capsid shell maturation [34].

The core DNA replication and repair machinery comprises six ORFs, including a DNA polymerase (ORF43), a DNA primase (ORF41), and a DNA helicase (ORF31), supplemented by auxiliary nucleases and processing enzymes, including an HNH endonuclease (ORF10), a putative polynucleotide kinase (ORF14), and a PD-(D/E)XK family nuclease (ORF36).

**Figure 4:**
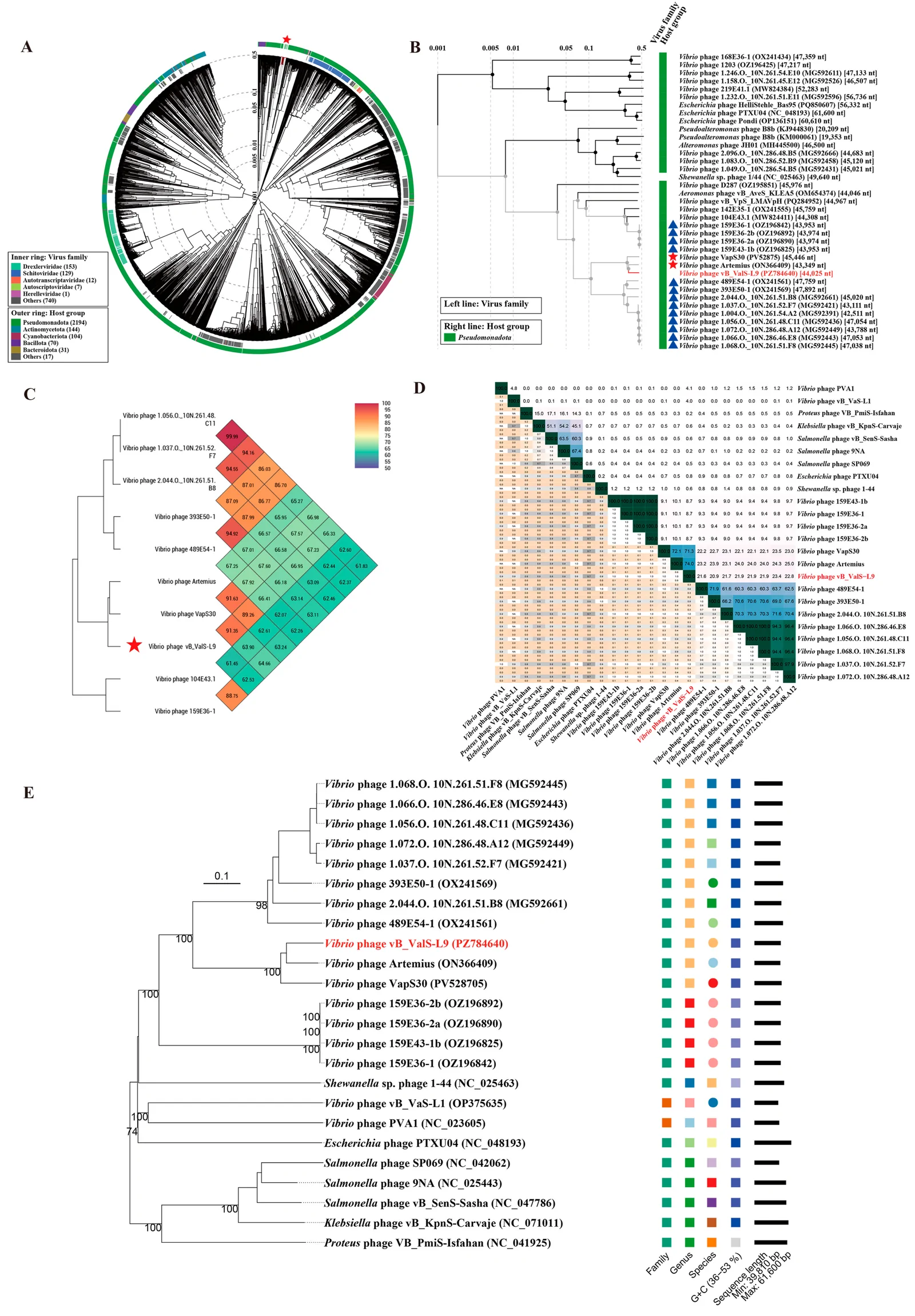
Phylogenomic and comparative genomic analysis of phage vB_ValS-L9 and its closely related *Vibrio* phages. (**A**) Proteome-based phylogenetic tree generated by VIPTree displaying the global genomic relationships between phage vB_ValS-L9 and related double-stranded DNA viruses in the NCBI database. The colored rings represent the virus family (inner ring) and host group (outer ring). The red star marks the position of phage vB_ValS-L9. (**B**) Expanded sub-tree from VIPTree detailing the clade containing phages vB_ValS-L9, VapS30, and Artemius and their closely related genomic neighbors. The left and right color bars indicate the taxonomic virus family and host group, respectively. The red stars indicate phages VapS30 and Artemius, while the blue triangles represent their closely related genomic neighbors. (**C**) Heatmap of Average Nucleotide Identity (ANI) values showing pairwise sequence comparison between phage vB_ValS-L9 and representative bacteriophages. The red star indicates phage vB_ValS-L9. (**D**) Heatmap of intergenomic similarities between phage vB_ValS-L9 and its closest relatives, calculated using the VIRIDIC pipeline. The upper-right half displays pairwise intergenomic identities (%), while the lower-left half represents aligned genome fractions. (**E**) Genome-based phylogenetic tree constructed using the VICTOR pipeline at the nucleotide level (D0), depicting the taxonomic classification and clade support values inferred using genome-BLAST distance phylogeny (GBDP).

Virion architecture is encoded by seven structural genes, including the major capsid protein (ORF57), neck protein (ORF50), stopper protein (ORF55), tail tubular protein (ORF44), tail tube protein (ORF52), tail length tape measure protein (TMP) (ORF49), and major tail protein (ORF54). Notably, TMP (ORF49) comprises 945 amino acids, corresponding to a predicted tail length of approximately 141.75 nm based on the 0.15 nm/residue ratio [35,36]. This estimate is consistent with the tail length measured by TEM (143.03 ± 5.32 nm) (Figure 1 and Figure 4).

To overcome host barriers and regulate gene expression, vB_ValS-L9 encodes two regulatory and counter-defense effectors: ORF9 (TreK) is predicted to act during early infection to shut down host macromolecular synthesis and redirect cellular metabolism toward viral replication (NCBI Protein Family Model NF049213, https://www.ncbi.nlm.nih.gov/genome/annotation_prok/evidence/NF049213/#1, accessed on 16 August 2026), whereas ORF22 encodes a restriction alleviation protein (Lar) that assists in overcoming host restriction-modification (R-M) barriers (Encyclopedia of Viral Anti-DefencE Systems, http://www.ebi.ac.uk/finn-srv/evades/details/lar/, accessed on 16 August 2026).

### 2.4. Comparative Genomics, Phylogenetic Classification, and Taxonomic Clustering

#### 2.4.1. Phylogenetic Classification, Comparative Genomics, and Evolutionary Divergence

Initial BLASTn searches against the NCBI nucleotide database, performed on 13 October 2026, revealed that the genome of phage vB_ValS-L9 showed more than 1% query coverage with 17 *Vibrio* phage genomes, of which only four were isolated phages (Table S3). Among these, phage vB_ValS-L9 showed the highest sequence similarity to two phages: *Vibrio* phage VapS30 (95.11% identity with 72% query coverage; accession no. PV528705) and *Vibrio* phage Artemius (95.81% identity with 74% query coverage; accession no. ON366409) [37]. The remaining 15 phages all showed no more than 6% query coverage with phage vB_ValS-L9. This indicated an exceptionally close evolutionary relationship among those three phages, prompting their triadic comparative analysis throughout subsequent genomic investigations.

To delineate their global evolutionary placement, a whole-genome proteomic tree was constructed via the ViPTree server, including all 17 homologous phages retrieved from NCBI (Figure 4A,B). Although phages vB_ValS-L9, VapS30, and Artemius clustered within the larger clade of *Vibrio* phages, they formed a tightly clustered, well-supported monophyletic clade that was separated from all other recognized *Vibrio*-infecting phages. Among these 17 related phages, their closest relatives outside this group were predominantly uncultured viruses. Based on the ViPTree topology and NCBI BLAST searches, a set of phylogenetically related phage genomes was selected for subsequent comparative analyses.

Subsequently, intergenomic pairwise comparisons using OrthoANI demonstrated that vB_ValS-L9 shares 91.35% average nucleotide identity (ANI) with VapS30, 89.26% with Artemius and less than 67.23% with other phages (Figure 4C). Because these values are above the 70% genus-level demarcation threshold but below the 95% species-level boundary [38], vB_ValS-L9 represents a novel viral species within the same genus as VapS30 and Artemius. Intergenomic similarity profiling via VIRIDIC yielded completely concordant clustering and boundaries (Figure 4D). Moreover, intergenomic similarities between vB_ValS-L9 and other phage genomes were all less than 23.4%.

**Figure 5:**
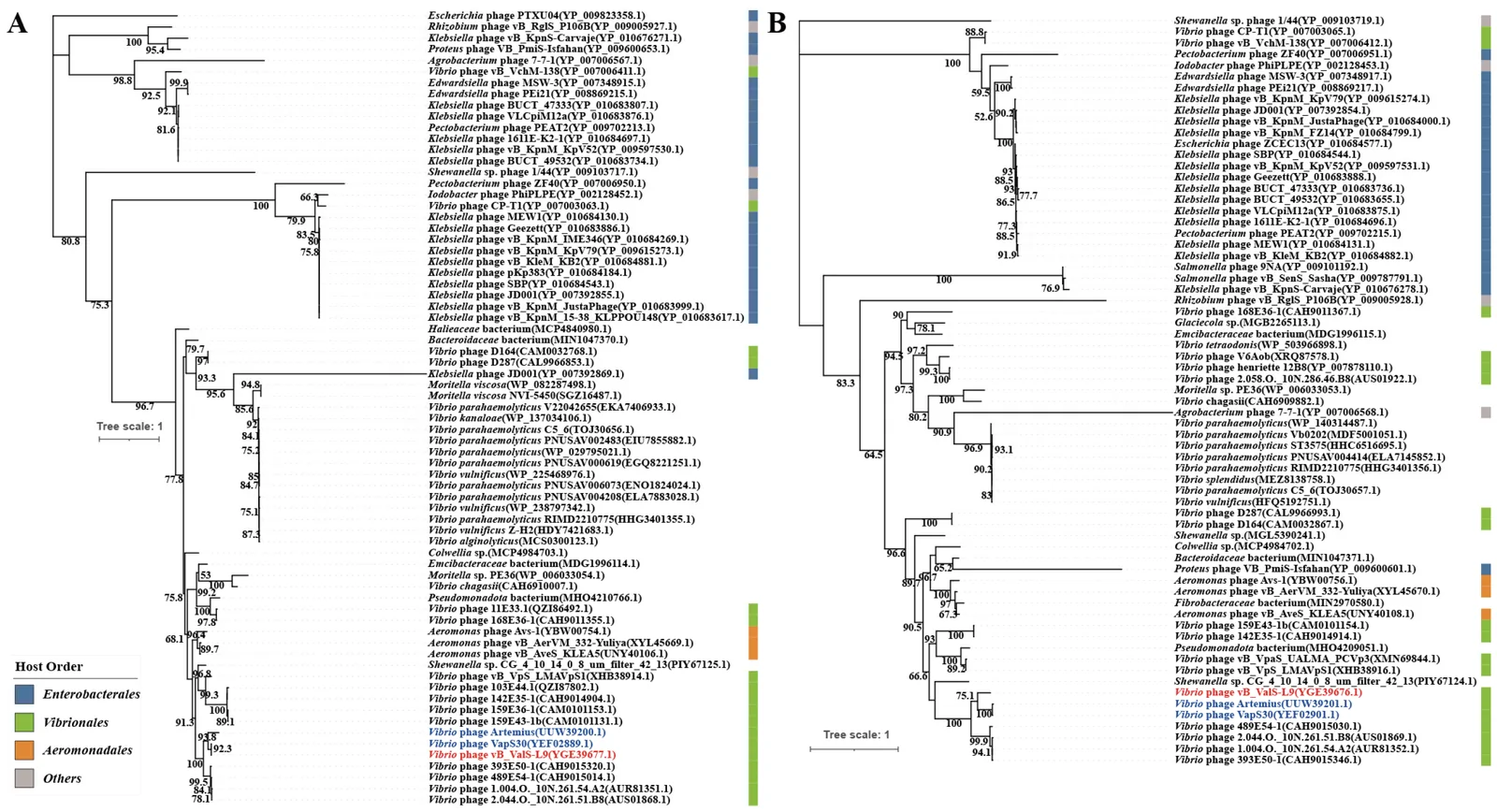
Maximum-likelihood (ML) phylogenetic analysis of phage vB_ValS-L9 based on conserved packaging proteins. (**A**) ML phylogenetic tree inferred from the amino acid sequences of the terminase. (**B**) ML phylogenetic tree constructed from the amino acid sequences of the portal protein. Branch support values were evaluated using 1000 ultrafast bootstrap replicates, and node values greater than 50% are indicated at the corresponding branches. Phage vB_ValS-L9 is shown in red, and phage Artemius and phage VapS30 are shown in blue.

Furthermore, a genome-BLAST distance phylogeny generated using the VICTOR pipeline robustly placed vB_ValS-L9, VapS30, and Artemius into an independent monophyletic lineage (Figure 4E). Single-gene maximum-likelihood (ML) phylogenetic trees reconstructed from the amino acid sequences of the terminase (ORF60) and portal protein (ORF59) further validated this topology (Figure 5), demonstrating that vB_ValS-L9 invariably coalesces into the same tight clade with VapS30 and Artemius with maximal bootstrap support. Moreover, these three phages formed a larger, well-supported clade with four uncultured viral genomes, namely *Vibrio* phage 489E54-1 (CAH9015014), *Vibrio* phage 393E50-1 (CAH9015320), *Vibrio* phage 1.004.O._10N.261.54.A2 (AUR81351), and *Vibrio* phage 2.044.O._10N.261.51.B8 (AUS01869). These four phages were assembled from seawater-derived sequence data; however, detailed information about them remains limited in the NCBI database and the published literature [39].

#### 2.4.2. Comparative Genomics and Orf-Level Variations Underlying Phenotypic Divergence

**Figure 6:**
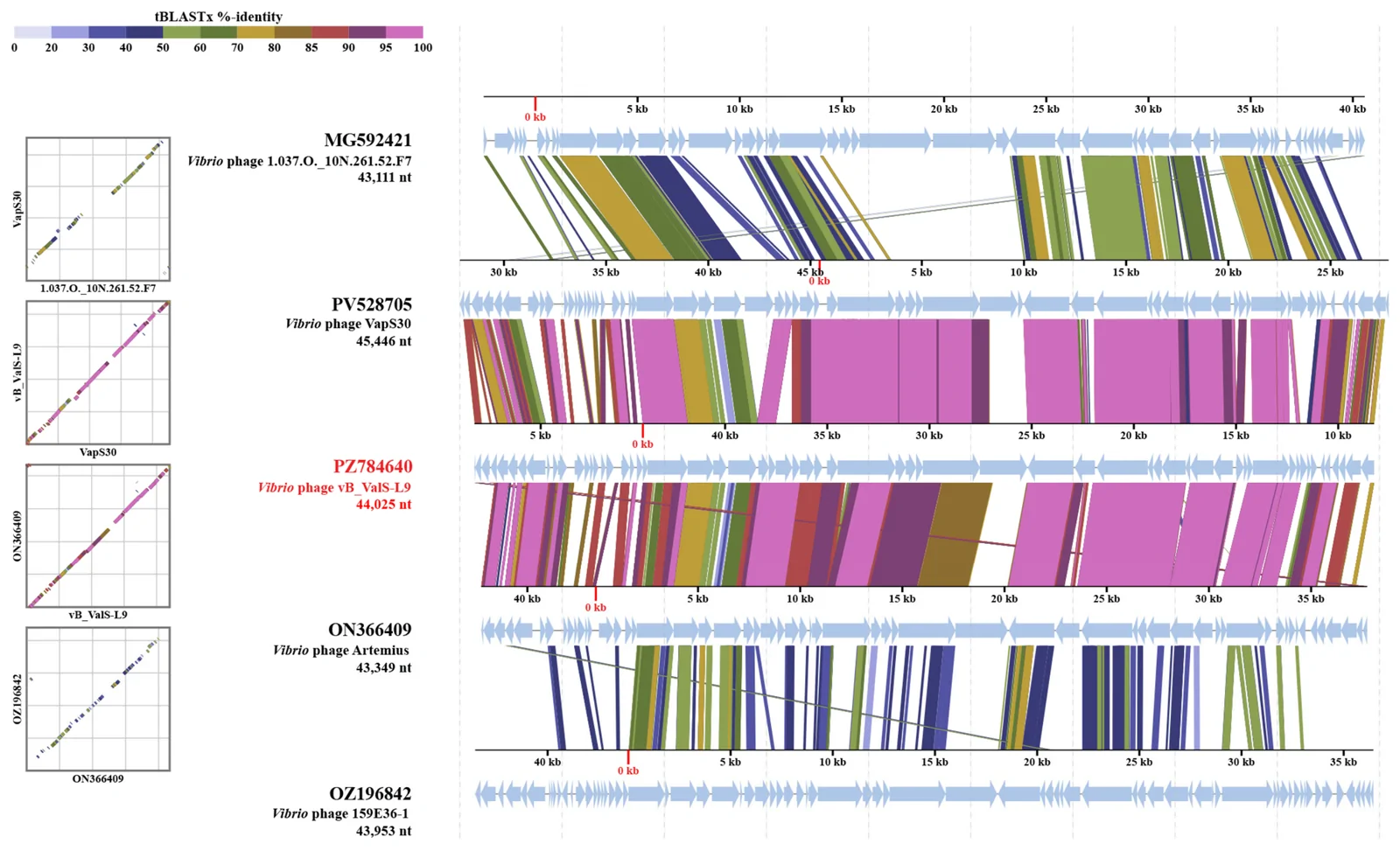
Genomic comparison between *Vibrio* phage vB_ValS-L9, VapS30, Artemius, 1.037.O._10N.261.52.F7, and 159E36-1. Arrows represent predicted ORFs, and their orientations indicate the direction of transcription. Genome regions showing similarity were identified using tBLASTX. Matches meeting the specified E-value threshold (<10^−5^) are indicated by colored blocks according to the scale above.

Pairwise genomic comparisons of vB_ValS-L9 with VapS30, Artemius [37], *Vibrio* phage 1.037.O._10N.261.52.F7 (hereafter F7, MG592421) [39], and *Vibrio* phage 159E36-1 (hereafter 159E36-1, OZ196842) performed using tBLASTx revealed high overall genomic synteny and modular conservation among the three vibriophages (Figure 6). A total of 47 ORFs (77.05% of the 61 predicted ORFs) in vB_ValS-L9 exhibited significant homology to genes in Artemius (amino acid identity: 30.56–100%), while 46 ORFs (75.41%) were homologous to those in VapS30 (amino acid identity: 35.23–100%) (Table S4). Furthermore, vB_ValS-L9 shared significant homology with phage F7 (30 ORFs; 29.05–74.82% identity) and phage 159E36-1 (26 ORFs; 26.83–61.00% identity), primarily involved in essential functional modules, including DNA replication and repair, structural components, and viral packaging.

Beyond the conserved core, distinct patterns of gene acquisition and loss were identified among the three closely related phages. Notably, ORF9 (the early host-takeover effector TreK) and ORF28 (endolysin) were unique to vB_ValS-L9, with no detectable homologs in either VapS30 or Artemius. Furthermore, ORF33 (a coiled-coil domain-containing protein) was exclusively present in vB_ValS-L9. Gene distribution differences were also observed across pairwise comparisons: ORF22 (the restriction alleviation protein Lar) and ORF34 (ATPase) were shared between vB_ValS-L9 and VapS30 (sharing 60.00% and 91.87% amino acid identity, respectively) but were completely absent in Artemius. Conversely, ORF30 (DUF4326 domain-containing protein) was conserved between vB_ValS-L9 and Artemius (100.00% identity) but missing in VapS30. In contrast, enzymatic genes such as ORF6 (cellulase) and ORF11 (hydrolase) were present in VapS30 but absent in both vB_ValS-L9 and Artemius.

In addition to gene presence/absence polymorphisms, structural sequence divergence was evident. Most notably, ORF44 (tail tubular protein), a structural component typically implicated in host cell attachment and penetration, displayed marked sequence divergence, exhibiting only 75.76% amino acid identity to its VapS30 counterpart and 75.37% to that of Artemius.

#### 2.4.3. Gene-Sharing Network Analysis and Taxonomic Clustering

**Figure 7:**
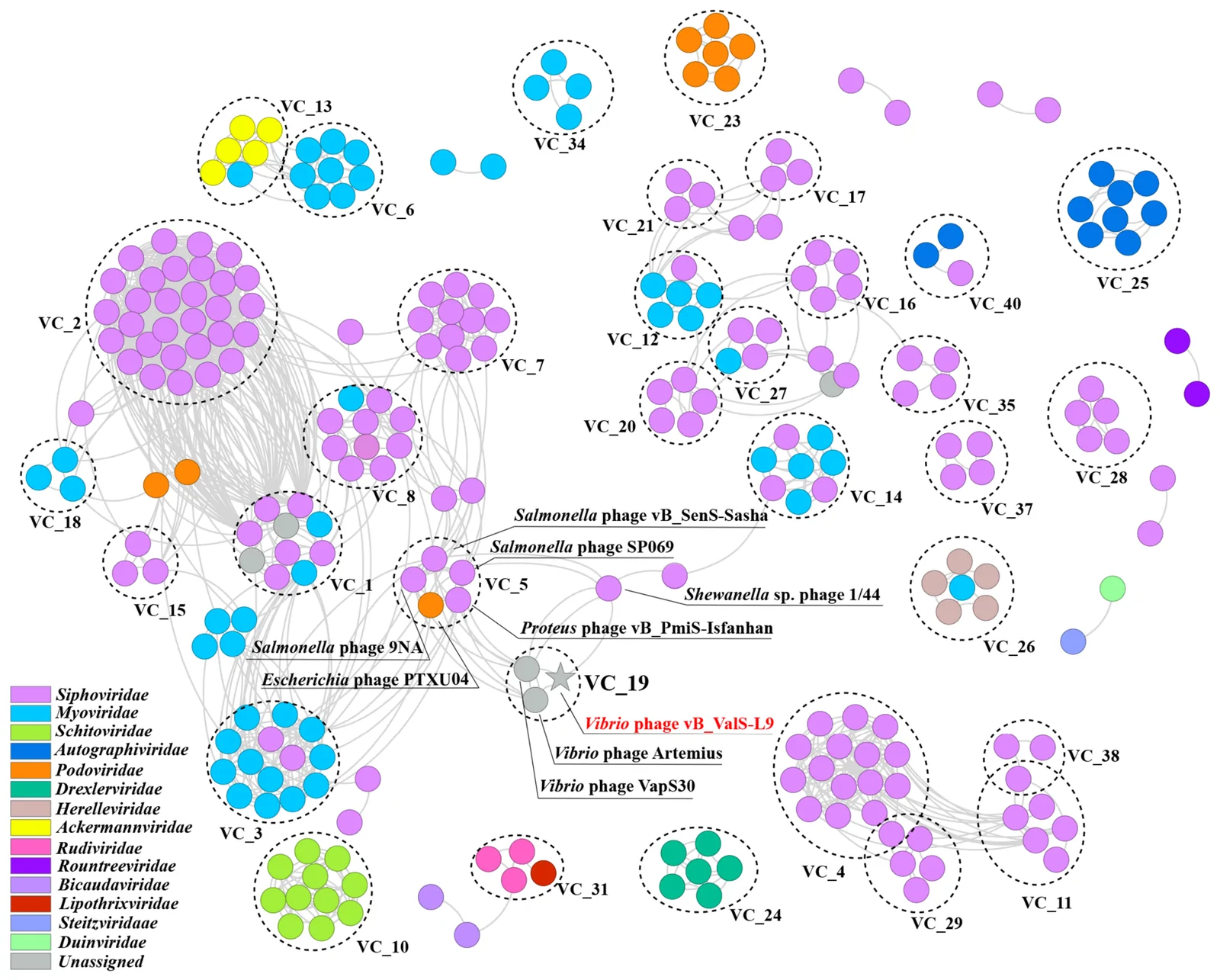
Gene-sharing network analysis of phage vB_ValS-L9, VapS30, Artemius, and related prokaryotic viruses from the NCBI RefSeq database. Nodes represent individual phage genomes, and edges between two nodes indicate their statistically weighted pairwise similarities with phage–phage similarity scores of ≥1. The network was visualized using Gephi version 0.9.7. Viruses at different family levels are shown in different colors. Dotted lines enclose different virus clusters. The star and red font highlight phage vB_ValS-L9 isolated in this study.

To further validate the taxonomic framework of vB_ValS-L9 at the viral community scale, a gene-sharing network analysis was performed using datasets from GenBank (*Coheviridae*, class *Caudoviricetes*). Network topology and density-based clustering using ClusterONE demonstrated that phage vB_ValS-L9 only exhibited linkages with phage VapS30, Artemius and *Shewanella* sp. phage 1/44 [40]. In contrast, VapS30 and Artemius showed linkages with six additional isolated phages, none of which are *Vibrio* phages. Among these, vB_ValS-L9, together with VapS30 and Artemius, grouped cohesively into a single distinct virus cluster (VC) module (VC_19), distinct from the other viral clusters (VCs) identified in the network (Figure 7). Although six additional phages were connected to members of VC_19, five were assigned to VC_5, whereas the remaining phage was unclustered. These findings provided additional evidence for the close genomic relationship among vB_ValS-L9, VapS30, and Artemius.

### 2.5. Protective and Therapeutic Efficacy of Phage Vb_vals-L9 in P. martensii Challenge Trials

**Figure 8:**
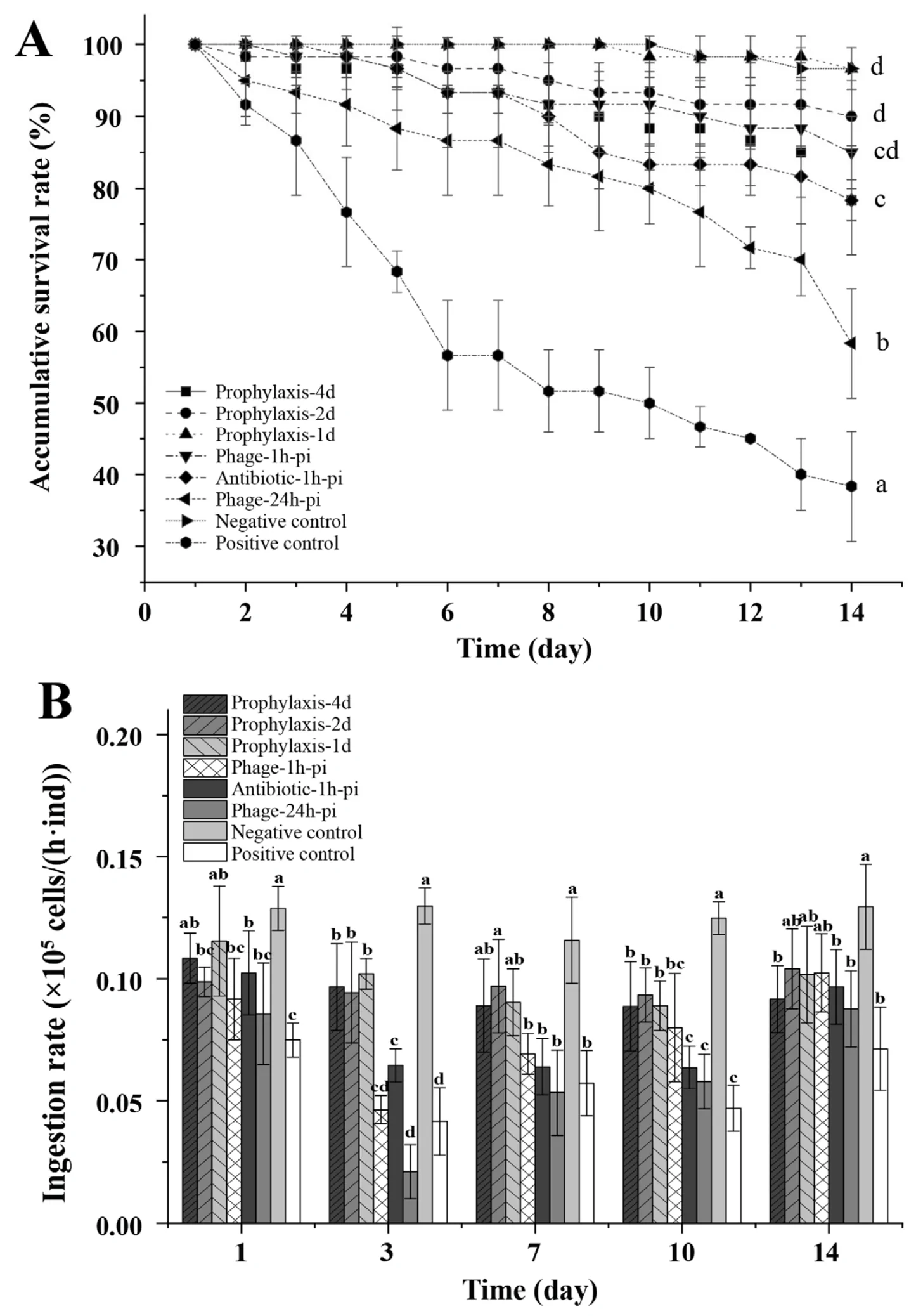
Prophylactic and therapeutic effects of phage vB_ValS-L9 on the survival and ingestion rates of juvenile pearl oysters (*Pinctada martensii*). (**A**) Accumulative survival of juvenile pearl oysters under different prophylactic and therapeutic treatment regimens during the 14-day experimental period. Final survival rates were compared among the eight groups using one-way analysis of variance (ANOVA). Statistically significant differences (*p* < 0.05) are indicated by different lowercase letters. (**B**) Ingestion rates of juvenile pearl oysters after bacterial challenge under the corresponding treatment regimens. Differences in ingestion rates among groups were analyzed separately at each sampling time point using one-way ANOVA. Statistically significant differences (*p* < 0.05) are indicated by different lowercase letters. Prophylaxis-4d, Prophylaxis-2d, and Prophylaxis-1d, refer to phage immersion 4, 2, and 1 d prior to *Vibrio* challenge, respectively; Phage-1h-pi and Phage-24h-pi, phage administration 1 h and 24 h post-challenge, respectively; Antibiotic-1h-pi, florfenicol treatment 1 h post-challenge; Negative control, unchallenged and untreated oysters; and Positive control, *V. alginolyticus* L4 challenge without treatment.

The in vivo biocontrol capacity of phage vB_ValS-L9 in mitigating *V. alginolyticus* L4-induced mortality in juvenile pearl oysters was assessed over a 14-day trial (Figure 8). In the untreated negative control group, oysters maintained high baseline viability throughout the experiment, exhibiting a 14-day cumulative survival rate of 96.67% (cumulative mortality: 3.33%). Conversely, oysters in the positive control group (challenged with *V. alginolyticus* L4 without intervention) experienced severe mortality beginning on day 2, with the survival rate declining steeply to 56.67% at day 7 and reaching a final survival of only 38.33% by day 14 (final mortality: 61.67%) (*p* < 0.001). Prophylactic phage administration conferred pronounced protective effects, with survival directly correlating with the proximity of phage delivery to the bacterial challenge. Among the prophylaxis groups, the Prophylaxis-1d group yielded the highest protection, achieving a final 14-day survival rate of 96.67% (comparable to the unchallenged negative control, *p* > 0.05). Prophylaxis-2d and Prophylaxis-4d groups also conferred substantial protection, yielding final survival rates of 90.00% and 78.33%, respectively, both significantly higher than the positive control (*p* < 0.01). In therapeutic regimens, the Phage-1h-pi group effectively rescued infected oysters, resulting in an 85.00% survival rate at day 14, which surpassed the survival rate of the Antibiotic-1h-pi group (+1 h antibiotic, 78.33% survival). Although delayed phage intervention at 24 h post-infection (+24 h) showed reduced efficacy due to advanced bacterial colonization and tissue injury, it still significantly enhanced juvenile survival (58.33%) relative to the positive control (38.33%, *p* < 0.05).

The effects of phage treatment on the physiological status of juvenile pearl oysters were also assessed by monitoring their ingestion rates (Figure 8B). Overall, the positive control group exhibited a pronounced reduction in ingestion activity following *V. alginolyticus* L4 challenge, with the lowest ingestion rate observed on day 3, followed by a partial recovery thereafter. In contrast, oysters receiving phage prophylaxis or early therapeutic treatment generally maintained higher and more stable ingestion rates throughout the 14-day experiment. The prophylactic groups showed relatively stable ingestion activity across the observation period, whereas the Phage-24h-pi group exhibited a more pronounced reduction in ingestion rate, particularly during the early and middle stages of infection. Notably, the ingestion rates of phage-treated oysters were generally comparable to those of the negative control and were higher than those of the positive control at several time points. These results indicate that phage treatment not only improved survival following *V. alginolyticus* challenge but also helped preserve the feeding activity of juvenile pearl oysters.

## 3. Discussion

### 3.1. Taxonomic Novelty and Genomic Characteristics of Phage Vb_vals-L9 and Its Relatives

The combined evidence from whole-genome proteomic trees, ANI, VIRIDIC, VICTOR, and single-gene phylogenies places phages vB_ValS-L9, VapS30, and Artemius in a well-supported monophyletic lineage that is clearly distinct from other known *Vibrio* phages [37]. The ANI values among these phages fall above the genus-level threshold but below the species-level boundary [38], justifying the designation of vB_ValS-L9 as a novel species within a new genus. We tentatively propose the name “*Artemiovirus*” for this genus. Gene-sharing network analysis further supports this grouping, as the three phages formed a cohesive virus cluster separated from established phage families (Figure 7). This taxonomic placement highlights the still-underexplored diversity of marine *Vibrio* phages and the need for continued isolation and genomic characterization.

### 3.2. Conserved and Lineage-Specific Genomic Features

Comparative genomics revealed a conserved core of genes involved in DNA replication, genome processing, and virion assembly, consistent with the close evolutionary relationship among the three phages. This core likely represents the functional backbone required for lytic replication in this lineage. In contrast, several genes showed lineage-specific distributions, including ORF9 (TreK), ORF28 (endolysin), and ORF33 in vB_ValS-L9; ORF22 (Lar) shared with VapS30; and ORF30 shared with Artemius. These patterns suggest that gene gain, loss, and retention have contributed to divergence after speciation.

Phage vB_ValS-L9 encodes five TM helix-containing proteins, and similar TM-rich proteins are present in VapS30 and Artemius, with amino acid sequence identities ranging from 52.63% to 99.57%. Multiple TM helices may enable functional division and coordination among holin/antiholin proteins with distinct topologies, allowing precise timing of lysis initiation and optimization of lysis efficiency [41,42]. In F1 cluster phages, two holin-like genes are required for efficient lysis and temporal control; deletion of either gene alone impairs lysis, and co-production causes growth arrest [42]. Phage SPP1 likewise requires two holin-like proteins for coordinated lysis [43]. The *Mycobacterium* phage D29 holin uses a C-terminal coiled-coil domain and basic residues to regulate oligomerization and pore formation [44]. Thus, the TM-rich lysis module of these three phages may confer fine-tuned regulation of host lysis and enhance their ability to counter host defenses.

Although phage vB_ValS-L9 exhibits quite low overall sequence similarity to phages F7 and 159E36-1 (Figure 4C,D and Figure 6), nine genes encoding structural proteins and assembly-related enzymes, together with five genes involved in DNA replication, are conserved across all three genomes (identity ranges from 36.35% to 73.61% and from 29.73% to 61.0%, respectively), exemplified by the tail length tape measure protein (36.35% versus F7 and 46.08% versus 159E36-1), helicase (63.33% and 41.63%, respectively), major capsid protein (52.02% and 51.41%, respectively), and DNA polymerase (56.71% and 48.18%, respectively) (Table S4). This relative conservation of a functionally diverse core amid substantial sequence divergence suggests that phages vB_ValS-L9, F7, and 159E36-1 share a common virion architecture and replication machinery, consistent with the modular genome organization of bacteriophages and supporting the existence of a core genome for essential virion formation [45].

### 3.3. Host Range, Tail-Associated Divergence, and Early Infection

Among the 53 bacterial strains tested (including 5 *Vibrio alginolyticus* strains), phage vB_ValS-L9 exhibited lytic activity only against its original host, indicating a relatively narrow host range among the strains tested, whereas phage Artemius infected 13 of the 20 tested *Vibrio* strains [37]. Tail-associated structural proteins are key determinants of host recognition. Notably, the tail tubular protein ORF44 of vB_ValS-L9 shares only 75.76% and 75.37% amino acid sequence identity with its counterparts in VapS30 and Artemius, respectively. The portal protein ORF59 also shows relatively low conservation, with 68.46% and 68.70% identity to Artemius and VapS30, respectively. In addition, the tail tube protein ORF52 is highly conserved with Artemius (96.55% identity) and VapS30 (98.71% identity), whereas the major capsid protein ORF57 exhibits only 52.41% and 52.63% identity with the corresponding proteins of VapS30 and Artemius, respectively. Collectively, despite a conserved overall genome organization, these three phages show substantial divergence in virion structural proteins, especially tail-associated components involved in host recognition and genome delivery, which may underlie their different host-interaction properties [46,47,48]. However, causality between these sequence differences and host specificity remains to be experimentally established. Expanding host-range testing to include more *V. alginolyticus* isolates from diverse sources will help determine whether the narrow host range of vB_ValS-L9 is an intrinsic property or an artifact of limited sampling.

Early infection requires phages to overcome host metabolic competition and defense systems [49,50,51]. Phage vB_ValS-L9 encodes TreK (ORF9), a putative early host-takeover effector that may suppress host macromolecular synthesis and redirect resources toward viral replication [52]. However, this gene is absent in the closely related phages VapS30 and Artemius. TreK homologs have been identified in several staphylococcal Twort-like phages, and their conservation suggests possible roles in host-range determination or genome packaging [53]. In phage SPO1, the early gene cluster within the terminal redundancy constitutes a typical “host-takeover module” comprising 24 genes organized into 12 operons, whose products inhibit host macromolecular synthesis to create favorable conditions for viral replication [54,55,56]. The presence of TreK only in vB_ValS-L9 among the three closely related phages suggests that it may contribute to its specific host-adaptation strategy. In addition, ORF22 encodes Lar, a restriction alleviation protein that likely helps the phage bypass host R-M barriers [57,58,59,60].

### 3.4. Prophylactic and Therapeutic Efficacy in Pearl Oysters

Vibriosis caused by *V. alginolyticus* remains a major constraint on the sustainable development of marine aquaculture [61]. In the juvenile *P. martensii* challenge model, vB_ValS-L9 conferred substantial prophylactic and therapeutic protection. The three prophylaxis groups and the Phage-1h-pi group achieved significantly higher 14-day survival rates than the positive control group, indicating that early administration provides superior protection. This finding is consistent with previous observations that prophylactic phage application generally outperforms delayed treatment in aquaculture systems [62,63]. Filter-feeding bivalves can bioaccumulate virus particles in digestive tissues, gills, and mantle [64].

The efficacy of prophylaxis declined when phage was administered 4 days before challenge compared with 2 days and 1 day before challenge, likely due to phage decay in seawater and clearance by the host. Nakai et al. found that phages survived for only 3 days in natural seawater [65]; Suttle and Chen reported decay rates of 0.4–0.8 h^−1^ under full sunlight [66]; and Suttle estimated an average daily decay of approximately 0.48, corresponding to about 38% inactivation per day [67]. On this basis, phage administered 4 days earlier would retain only a fraction of the titer of that administered 1 day earlier, making it difficult to maintain an effective concentration at the time of infection. In addition, bivalve hemolymph and phagocytes can clear phages [68,69]. Therefore, the prophylactic effect follows an inverted U-shaped relationship with administration time, peaking at approximately 1 day, suggesting that shorter intervals or repeated dosing may be preferable in practice.

The Phage-24h-pi group showed lower efficacy than the early intervention groups, likely related to deep bacterial colonization and tissue damage at later stages. Lomelí-Ortega and Martínez-Díaz showed that phage therapy within 6 h post-infection effectively prevented mortality of *Litopenaeus vannamei* larvae, whereas administration after 6 h still reduced mortality but was less effective than early intervention [70]. The reduced efficacy of delayed administration may be related to the difficulty of phages reaching infected tissues such as the hepatopancreas [70]. Alternatively, bacteria may have already begun to colonize or invade pearl oyster cells [71], and phages may need to compete with established bacterial communities for adsorption sites [72]. Therefore, the optimal window for phage therapy should be early infection or prophylactic use. Notably, the Phage-1h-pi and Antibiotic-1h-pi groups produced similar protective effects, indicating that phage therapy is comparable or superior to antibiotic treatment.

Furthermore, the vB_ValS-L9 genome contains no tRNA genes, virulence factors, antibiotic resistance genes, or lysogeny-associated markers (integrases, recombinases, and excisionases), consistent with its strictly lytic lifestyle and supporting its safety as a biocontrol agent [73]. Its narrow host range may minimize disturbance to non-target microbial communities and protect beneficial bacteria in aquaculture environments [23,61], but it also limits its applicability as a broad-spectrum antimicrobial. Phage cocktail strategies can increase bacterial coverage and reduce the probability of resistance emergence [61,74]. Therefore, vB_ValS-L9 may need to be combined with phages targeting other *V. alginolyticus* strains.

## 4. Materials and Methods

### 4.1. Bacterial Strains and Culture Conditions

*Vibrio alginolyticus* L4, originally isolated from pearl oyster (*Pinctada martensii*) tissues sampled from an aquaculture site on Weizhou Island (Beihai, China), was employed as the host bacterium for bacteriophage isolation and propagation. In addition, 53 bacterial strains were utilized to evaluate the phage host range (Table S1). All bacterial strains were routinely grown in 2216E marine broth with shaking at 180 rpm or maintained on 2216E marine agar plates (Hope Bio-Technology Co., Ltd., Qingdao, China) at 28 °C. The 16S rRNA gene sequence of *Vibrio alginolyticus* L4 was submitted to GenBank under accession number PZ766330.

### 4.2. Phage Isolation, Purification, and Biological Characterization

#### 4.2.1. Phage Isolation and Purification

Water samples were collected from the seawater in the coastal aquaculture area in Beihai, China (21.46° N, 109.35° E). Samples were clarified by low-speed centrifugation and passed through 0.22 μm membrane filters (Millipore, Bedford, MA, USA) to remove cellular debris and bacteria. To enrich for bacteriophages, the resulting filtrate was mixed with an equal volume of early logarithmic-phase *Vibrio alginolyticus* L4 culture in 2216E broth and incubated at 28 °C. After 24 and 48 h of incubation, the enrichment mixtures were centrifuged at 6000× *g* for 10 min at 4 °C. The supernatants were filtered through 0.22 μm membrane filters and screened for lytic phages using the double-layer agar method [75]. Single plaques were collected in sodium chloride-magnesium sulfate (SM) buffer (100 mM NaCl, 50 mM Tris, 10 mM MgSO_4_, and 0.01% [*w*/*v*] gelatin, at pH 7.5) and subjected to five consecutive rounds of plaque purification to obtain a clonal isolate. The purified phage was suspended in SM buffer and stored at 4 °C for routine experiments, with long-term stocks supplemented with chloroform at −80 °C.

#### 4.2.2. Chloroform Sensitivity Test

To determine whether the phage possesses a lipid-containing envelope or membrane components, its sensitivity to chloroform was evaluated following the protocol described by Lu et al. [76]. Briefly, 500 µL aliquots of purified phage suspension (~10^7^ PFU/mL) were mixed with 5, 50, or 500 µL of chloroform (corresponding to final volume fractions of 1%, 10%, and 50%, respectively). A phage suspension without chloroform served as the negative control. The mixture was vortexed vigorously for 2 min and incubated at 28 °C for 30 min. After incubation, the samples were centrifuged at 4000× *g* for 5 min at 4 °C to separate the organic and aqueous phases. The upper aqueous phase containing active phages was carefully collected, serially 10-fold diluted, and titrated using the double-layer agar method. All assays were performed in triplicate.

#### 4.2.3. Transmission Electron Microscopy (TEM) and Morphological Analysis

To observe virion morphology, 1 L of phage lysate was clarified through a 0.22 μm membrane and precipitated by adding 10% (*w*/*v*) polyethylene glycol (PEG) 8000 supplemented with 1 M NaCl. Following overnight incubation at 4 °C, phage particles were pelleted by centrifugation at 10,000× *g* for 50 min at 4 °C and gently resuspended in 10 mL of SM buffer. Phage particles were further purified via CsCl isopycnic gradient ultracentrifugation (*ρ* = 1.5 g/mL) at 200,000× *g* for 24 h at 4 °C using an Optima L-100 XP Ultracentrifuge (Beckman Coulter, Brea, CA, USA). The purified phage band was harvested using a sterile syringe and dialyzed twice against SM buffer (4 °C, 12 h per exchange) to completely remove residual CsCl.

For grid preparation, 10 µL of the purified phage suspension was deposited onto a carbon-coated copper grid (200-mesh) and allowed to adsorb for 10 min. Excess liquid was blotted with filter paper, and the sample was negatively stained with 2% (*w*/*v*) aqueous uranyl acetate (pH 4.5) for 1 min, followed by air-drying. Specimen grids were examined using a JEOL JEM-1200EX transmission electron microscope (JEOL Ltd., Akishima, Tokyo, Japan) operating at 100 kV. Micrographs were recorded using a GATAN CCD camera system (Gatan, Inc., Pleasanton, CA, USA). Phage dimensions (head diameter and tail length) were determined by measuring at least 15 randomly selected, intact virions across five independent fields of view.

#### 4.2.4. Host Range Determination

To determine the infective host range of the isolated phage, spot assays were conducted on 53 distinct bacterial strains, including 43 *Vibrio* strains, seven *Shewanella* strains, two *Pseudoalteromonas* strains, and one *Sulfitobacter* strain (Table S1). Briefly, 5 μL aliquots of serially diluted phage suspensions (10^6^, 10^7^, and 10^8^ PFU/mL) were carefully spotted onto the surface of freshly prepared double-layer agar plates pre-inoculated with the respective test bacterium in the top agar. Following absorption of the droplets into the agar matrix, the plates were incubated at 28 °C for up to 7 days. Plates were monitored periodically for the formation of distinct lysis zones or individual plaques, which indicated susceptibility to phage infection. All spot assays were performed in triplicate.

#### 4.2.5. One-Step Growth Curve Assay

A one-step growth assay was performed to determine the latent period, eclipse period, and burst size of the phage, following established protocols [75,77] with minor modifications. Briefly, an exponentially growing culture of *V. alginolyticus* L4 (OD_600_ = 0.2, ~1 × 10^8^ CFU/mL) was infected with the phage suspension at a multiplicity of infection (MOI) of 0.01. The phage–host mixture was incubated at 28 °C for 10 min to facilitate viral adsorption, followed by centrifugation at 6000× *g* for 5 min at 4 °C to remove unadsorbed phages. The pellet of infected bacterial cells was gently resuspended in 2216E liquid medium and continuously incubated at 28 °C.

Samples were collected in triplicate at 10 min intervals over a 60 min period. To monitor intracellular phage progeny (determining the eclipse phase), a parallel set of aliquots was treated with chloroform (1% final volume fraction) to artificially lyse the cells. Both untreated and chloroform-treated samples were immediately diluted serially and titrated using the double-layer agar plaque assay. Bacterial growth was simultaneously monitored by recording the OD_600_. The burst size was calculated as the ratio of the final titer of liberated progeny virions to the initial quantity of infected bacterial cells during the latent period. All assays were conducted in three independent biological replicates.

### 4.3. Thermal, pH, and Salinity Stability Assays

To assess the environmental resilience of phage vB_ValS-L9, its tolerance to variations in temperature, pH, and salinity was evaluated in parallel using phage suspensions at ~10^8^ PFU/mL in SM buffer. Thermal stability was determined by incubating phage suspensions at temperatures ranging from 4 to 40 °C for 24 h, followed by immediate cooling to 4 °C and titration via the double-layer agar method. For the pH stability assay, the pH of 0.2 M Na_2_HPO_4_/NaH_2_PO_4_ buffered SM solutions was adjusted to values ranging from 5.0 to 10.0 using 5 M HCl or NaOH, followed by filtration through a 0.22 μm membrane (Millipore, Bedford, MA, USA). Aliquots of phage suspension were mixed with 950 μL of the respective pH-adjusted buffer and incubated at 28 °C for 24 h prior to plaque enumeration. Salinity tolerance was similarly examined by inoculating 50 µL of phage suspension into 950 µL of sterile seawater matrices with salinities ranging from 0‰ to 34‰ (prepared by serially diluting 34‰ sterile seawater with sterile distilled water), followed by 24 h incubation at 28 °C and subsequent plaque assay.

### 4.4. Genomic Sequencing, Annotation, and Phylogenetic Analysis

#### 4.4.1. Phage DNA Extraction and Genome Sequencing

Phage genomic DNA was extracted using the standard phenol–chloroform protocol [78] with minor modifications. Briefly, CsCl-purified phage particles were pretreated with DNase I (1 μg/mL) and RNase A (1 μg/mL; Takara Bio Inc., Kusatsu, Shiga, Japan) at 37 °C for 1 h to digest residual host genomic DNA and RNA. The mixture was subsequently incubated with proteinase K (100 μg/mL) in the presence of sodium dodecyl sulfate (SDS, final concentration: 0.5%, *w*/*v*) at 56 °C for 2 h to disrupt the viral capsid. Genomic DNA was isolated by sequential extractions with equal volumes of phenol:chloroform:isoamyl alcohol (25:24:1, *v*/*v*/*v*) and chloroform:isoamyl alcohol (24:1, *v*/*v*), precipitated with ice-cold isopropanol, washed twice with 70% ethanol, and finally dissolved in sterile TE buffer (pH 8.0). The purity and concentration of the extracted DNA were determined using a NanoDrop ND-1000 spectrophotometer (Thermo Fisher Scientific, Waltham, MA, USA) and verified by 1% (*w*/*v*) agarose gel electrophoresis.

High-throughput sequencing was conducted on an Illumina NovaSeq 6000 platform (Illumina, Inc., San Diego, CA, USA) with paired-end sequencing (2 × 150 bp) by Shanghai Biozeron Biotechnology Co., Ltd. (Shanghai, China). Raw reads were quality-filtered and adapter-trimmed using Trimmomatic (v0.36) with parameters SLIDINGWINDOW:4:15 and MINLEN:75 [79]. Genome assembly of the clean reads was performed using SPAdes (v3.15.5) under default multi-k-mer modes [80]. Genome completeness, potential host contamination, and circularity were evaluated using CheckV (v1.0.1) [81]. The complete genome sequence of phage vB_ValS-L9 has been deposited in the GenBank database under the accession number PZ784640.

#### 4.4.2. Genome Annotation and Bioinformatic Analysis

Putative open reading frames (ORFs) were predicted by integrating GeneMarkS (https://genemark.bme.gatech.edu/GeneMark/genemarks.cgi, accessed on 21 April 2026) [82] and PRODIGAL v2.6.3 [83]. Functional annotation was performed via GenomeNet nr-aa database (a non-redundant protein sequence database merging sequences from RefSeq, SwissProt, TrEMBL, and GenPept), with a cut-off E-value of 10^−5^ [84]. Remote homology searches and structural domain identification were performed using HHpred (https://toolkit.tuebingen.mpg.de/tools/hhpred, accessed on 21 April 2026) against PDB, Pfam, and SwissProt databases [85,86]. Putative tRNA genes were screened using tRNAscan-SE v1.23 [87,88]. To evaluate biosafety and lifestyle, predicted ORFs were queried against the Virulence Factor Database (VFDB) [89] and the Comprehensive Antibiotic Resistance Database (CARD) [90] using local BLASTp (BLAST+ version 2.16.0; E-value ≤ 10^−5^). Hallmark lysogenic markers (e.g., transposases, integrases, and recombinases) were systematically checked to confirm a lytic lifestyle [91,92]. A circular genome map was generated using the Proksee web server (https://proksee.ca/) [93].

#### 4.4.3. Comparative Genomics and Phylogenetic Classification

BLASTn searches were performed using the genome of the isolated phage as a query against the NCBI nr database, identifying 17 related viral genomes. A whole-genome proteomic tree was constructed using the Viral Proteomic Tree server (ViPTree, https://www.genome.jp/viptree/, accessed on 13 October 2026) based on normalized tBLASTx scores using all of the genomes [84]. Intergenomic pairwise similarities and nucleotide identities were computed using OrthoANI (OAT v0.93) [94] and the VIRIDIC web server (https://rhea.icbm.uni-oldenburg.de/VIRIDIC/) [95]. Genome-BLAST distance phylogeny was inferred via the Virus Classification and Tree Building Online Resource (VICTOR; https://ggdc.dsmz.de/victor.php) pipeline [96]. Single-gene maximum-likelihood (ML) phylogenetic trees were constructed for the amino acid sequences of the terminase (ORF60) and portal protein (ORF59) using IQ-TREE v3.0.1 [97] with 1000 ultrafast bootstrap replicates. The corresponding sequences were obtained from homologs identified by ViPTree topology and NCBI BLASTp searches (https://blast.ncbi.nlm.nih.gov/Blast.cgi, accessed on 14 October 2026). Phylogenetic trees were annotated and rendered using iTOL v7.6. Pairwise ORF-level amino acid synteny and similarities were analyzed using local BLASTp (BLAST+ version 2.16.0) with a cut-off E-value of 10^−5^ [98].

To further investigate the genomic relationships and taxonomic clustering of phage vB_ValS-L9 and related viruses, a gene-sharing network was constructed using vConTACT2 [99] with a reference dataset comprising 4534 prokaryotic viral genomes from NCBI Refseq (v211). Protein clusters (PCs) were generated using the Markov Cluster Algorithm (MCL), and viral clusters (VCs) were identified using the ClusterONE algorithm [100]. The protein-clustering E-value threshold was set to 1 × 10^−5^, and the maximum overlap parameter for viral cluster identification was set to 0.01. All other parameters were retained at their default settings, and the analysis was performed using eight computational threads. The resulting protein-sharing network was visualized using Gephi v0.9.7 [101].

### 4.5. In Vivo Potential Biocontrol Efficacy in Pearl Oysters

Juvenile pearl oysters (*Pinctada martensii*, shell length: 4.5 ± 0.3 cm) were acclimatized in recirculating seawater systems (temperature: 20 ± 1 °C, salinity: 30 ± 1, and pH: 8.0 ± 0.2) for 7 days prior to experimentation. Oysters were fed daily with *Chlorella vulgaris*, and half of the rearing seawater was renewed daily at 09:00 AM. To evaluate the prophylactic and therapeutic potential of phage vB_ValS-L9 against *V. alginolyticus* L4 infection, eight experimental groups were established in triplicate (*n* = 20 individuals per replicate, total 60 oysters per group) (Table 1). These included three phage prophylaxis groups (at Immunization Stage), in which a single phage dose was administered at 4, 2, or 1 day before bacterial challenge (Prophylaxis-4d, Prophylaxis-2d, and Prophylaxis-1d, respectively), with no further phage added during the 14-day *Vibrio* challenge period; two phage post-challenge groups, in which phage was added at 1 h and 24 h after *Vibrio* addition (Phage-1h-pi and Phage-24h-pi, respectively); one antibiotic group, in which florfenicol was added at 1 h after *Vibrio* addition (Antibiotic-1h-pi); one negative control (no phage and no *Vibrio*); and one positive control (*Vibrio* only).

For groups receiving bacterial challenge, immersion challenge was performed by inoculating mid-log-phase *V. alginolyticus* L4 into the culture water to achieve a final density of ~1.0 × 10^6^ CFU/mL. In phage-treated groups, phage was administered to a final concentration of ~1.0 × 10^6^ PFU/mL according to the designated schedule. Cumulative mortality was monitored, and samples were collected daily over a 14-day observation period. Ingestion rates of juvenile pearl oysters were measured on days 1, 3, 7, 10, and 14 after bacterial challenge for each corresponding treatment regimen. Dead individuals were promptly recorded and removed.

## 5. Conclusions

In summary, this study reports the isolation and comprehensive characterization of a novel lytic phage, vB_ValS-L9, infecting *V. alginolyticus*. The phage exhibits a siphovirus-like morphology, a circular double-stranded DNA genome of 44,025 bp with 61 ORFs, and remarkable stability across diverse environmental conditions. Comparative genomic and phylogenetic analyses indicate that vB_ValS-L9 represents a distinct phage species and, together with VapS30 and Artemius, may constitute a previously under-recognized genus-level lineage, provisionally designated *Artemiovirus*. Its narrow host range, environmental robustness, absence of virulence, antibiotic resistance, or lysogeny-associated genes, and significant in vivo prophylactic and therapeutic efficacy collectively indicate that vB_ValS-L9 is a safe and effective candidate for phage-based biocontrol of *V. alginolyticus* in aquaculture. This study expands the understanding of *Vibrio* phage diversity and provides a promising resource for sustainable aquaculture disease management.

## Figures and Tables

**Table 1:** Experiment design for phage therapy on pearl oysters.

Groups	Acclimation Stage	Immunization Stage	Inoculation Stage
	Day 1 to Day 6	3 days	14 days
Prophylaxis-4d	Add phage 4 days in advance	Phage was added via immersion at a final concentration of 1.0 × 10^6^ PFU/mL 4 days prior to the bacterial challenge	*Vibrio* was added daily via immersion to a final concentration of 1.0 × 10^6^ CFU/mL without phage supplementation.
Prophylaxis-2d	Add phage 2 days in advance	Phage was added via immersion at a final concentration of 1.0 × 10^6^ PFU/mL 2 days prior to the bacterial challenge	*Vibrio* was added daily via immersion to a final concentration of 1.0 × 10^6^ CFU/mL without phage supplementation.
Prophylaxis-1d	Add phage 1 day in advance	Phage was added via immersion at a final concentration of 1.0 × 10^6^ PFU/mL 1 day prior to the bacterial challenge	*Vibrio* was added daily via immersion to a final concentration of 1.0 × 10^6^ CFU/mL without phage supplementation.
Phage-1h-pi	Add the phage one hour after adding the *Vibrio*.	-	*Vibrio* was added daily via immersion to a final concentration of 1.0 × 10^6^ CFU/mL; 1 h later, phages were added to a final concentration of 1.0 × 10^6^ PFU/mL.
Antibiotic-1h-pi	Add antibiotics one hour after adding the *Vibrio*	-	*Vibrio* was added daily via immersion to a final concentration of 1.0 × 10^6^ CFU/mL; 1 h later, antibiotics (florfenicol) were added.
Phage-24h-pi	Add the phage 24 h after adding the *Vibrio*.	-	*Vibrio* was added daily via immersion to a final concentration of 1.0 × 10^6^ CFU/mL; 24 h later, phages were added to a final concentration of 1.0 × 10^6^ PFU/mL.
Negative control	Negative control	−	-
Positive control	Positive control	−	*Vibrio* was added daily via immersion to a final concentration of 1.0 × 10^6^ CFU/mL.

## Data Availability

The complete genomic sequence of phage vB_ValS-L9 has been deposited in GenBank under accession number PZ784640. The 16S rRNA gene sequence of Vibrio alginolyticus L4 has been deposited into GenBank under accession number PZ766330.

## Supplementary Materials

The following supporting information can be downloaded at: https://www.mdpi.com/article/10.3390/ijms27209385/s1. References [18,102,103] are cited in the Supplementary Materials.
