# Supplementary material for: Characterization and Genome Analysis of a Novel Vibrio Alginolyticus Phage and Its Biocontrol Efficacy in Pearl Oyster Aquaculture
Source: Int J Mol Sci. 2026 Oct 21;27(20):9385. doi: 10.3390/ijms27209385 (PMC13705570; doi:10.3390/ijms27209385)
Supplement: Supplementary file 1 [file ijms-27-09385-s001.zip › ijms-4608194-supplementary.pdf]

Table S1. Host range of phage vB_VasP-L10.

Strains	Best-matched species (% Id of 16S rDNA)	Source and location	Phage vB_VasP-L10	Ref.
L4	*Vibrio alginolyticus* strain I1B (99.78%)	Cells of *Pinctada fucata*, China	+	This study
ScY-2108	*Vibrio sanggoullaii* ScY-2108 (100.00%)	Cells of *Azumapecten farreri*, China	-	[1]
ABY-1904	*V. alginolyticus* ABY-1904 (100.00%)	Cells of *Haliotis discus hannai* Ino, China	-	[2, 3]
ScY-2109	*V. Sanggoullbii* ScY-2109 (100.00%)	Cells of *Azumapecten farreri,* China	-	[2, 3]
AbY-1805	*V. natriegens* ATCC 14048 (99.93%)	Cells of *Haliotis discus hannai* Ino, China	-	[2]
AbY-1905	*V. mediterranei* AbY-1905 (100.00%)	Cells of *Haliotis discus hannai* Ino, China	-	[2, 3]
AbY-2104	*V. mediterranei* AbY-2104 (100.00%)	Cells of *Haliotis discus hannai* Ino, China	-	[2, 3]
ScY-2106	*V. alginolyticus* ScY-2106 (100.00%)	Cells of *Azumapecten farreri,* China	-	[2, 3]
WaG-2229	*V. alginolyticus* WaG-2229 (100.00%)	Larval rearing water of *Haliotis discus hannai* Ino, China	-	[2, 3]
ScG-2111	*V. celticus* (100.00)	Cells of *Chlamys farreri*, China	-	[2]
ScY-2105	*V. alginolyticus* (99.72)	Cells of *Mizuhopecten yessoensis*, China	-	[2]
ScG-2110	*V. atlanticus* (99.92)	Cells of *Chlamys farreri*, China	-	[2]
ScY-2204	*V. splendidus* (99.93)	Cells of *Azumapecten farreri*, China	-	[2]
ScG-2206	*V. splendidus* (100.00)	Cells of *Azumapecten farreri*, China	-	[2]
AbY-1901	*V. owensii* (100.00)	Cells of *haliotis discus hannai ino*, China	-	[2]
AbY-1902	*V. mediterranei* (99.86)	Cells of *haliotis discus hannai ino*, China	-	[2]
AbG-1906	*V. chagasii* (100.00)	Cells of *haliotis discus hannai ino*, China	-	[2]

AbG-1907	*V. chagasii* (100.00)	Cells of *haliotis discus hannai ino*, China	-	[2]
Wa-1801	*Idiomarina sp.* (99.93)	Surface seawater, Yellow Sea, China	-	[2]
WaY-2101	*V. chagasii* (99.51)	Larval rearing water, China	-	[2]
WaY-2220	*V. alginolyticus* (99.72)	Larval rearing water, China	-	[2]
WaY-2207	*V. splendidus* (100.00)	Coastal seawater, China	-	[2]
L6	*V. owensii* strain YS03 (100%)	Larval rearing water of shrimp, China	-	This study
L7	*V. mediterranei* strain 3-25 (99.86%)	Larval rearing water of shrimp, China	-	This study
L8	*V. parahaemolyticus* strain 2012V-1165 (99.79%)	Cells of *Pinctada martensi*, China	-	This study
L11	*V. natriegens* strain G1 (99.93%)	Cells of *Crassostrea gigas*, China	-	This study
L12	*V. campbellii* strain DS1907-1YC1-1 (100.00%)	Coastal seawater of Beihai, China	-	This study
L37	*V. diabolicus strain* SF42 (99.81%)	Shrimp farm water, China	-	This study
L38	*V. campbellii* strain 2023 （99.72%））	Cells of *Trachinotus ovatus*, China	-	This study
L39	*V. harveyi* strain 6-16 (99.79%)	Cells of *Trachinotus ovatus*, China	-	This study
L41	*V. fortis* strain 20-1-10 (99.79%)	Culture seawater of coral, China	-	This study
L42	*V. ponticus* strain HAINUV03 (99.93%)	Culture seawater of coral, China	-	This study
L45	*V. harveyi* strain S0908 (99.79%)	Culture water of *Pinctada martensi*, China	-	This study
L48	*V. owensii* strain 20160513VC2W (100.00%)	Coastal seawater and *Pinctada margaritifera* seep water of Weizhou Island, China	-	This study
L49	*V. diabolicus* strain LMG 3418 (99.93%)	Coastal seawater and *Pinctada margaritifera* seep water of Weizhou Island, China	-	This study

L50	*V. alginolyticus* strain SK2.2 (99.86%)	Coastal seawater and *Pinctada margaritifera* seep water of Weizhou Island, China	-	This study
L55	*V. campbellii* strain 2023 (99.86%)	Culture seawater of coral, China	-	This study
C2*	*V. coralliilyticus strain* O2 (99.86%)	Coral, China	-	
MCCC1A14388*	*V. natriegens* (99.93%)	Shrimp farm water, China	-	
MCCC1K03861*	*V. natriegens* (99.93)	Sediment of oyster farming area, China	-	
MCCC 1H000251*	*V. natriegens* (100%)	Type strain	-	
ATCC17802*	*V. parahaemolyticus* (100.00%)	Type strain	-	
ScG-2112	*Shewanella japonica* (99.93)	Cells of *Chlamys farreri*, China	-	[2]
ScG-2113	*S. colwelliana* (100.00)	Cells of *Chlamys farreri*, China	-	[2]
AbG-1908	*S. waksmanii* (99.93)	Cells of *haliotis discus hannai ino*, China	-	[2]
AbG-2103	*S. japonica* (100.00)	Cells of *haliotis discus hannai ino*, China	-	[2]
WaG-2102	*S. japonica* (99.93)	Larval rearing water, China	-	[2]
L16	*S. colwelliana* strain HQN-8 (100.00%)	Cells of wild *Pinctada maxima*， China	-	This study
L18	*S. rhizosphaerae* FJAT-53764 (100.00%)	Coastal seawater of Beihai, China	-	This study
Wa-1802	*Pseudoalteromonas sp.* (100.00)	Surface seawater, Yellow Sea, China	-	[2]
Wa-1803	*P. marina* (99.93)	Surface seawater, Yellow Sea, China	-	[2]
Wa-1804	*Sulfitobacter sp.* (99.97)	Larval rearing water, China	-	[2]

[a] Purchased from the Marine Culture Collection of China, Xiamen, China.

Table S2. Genome annotation of phage vB_VasP-L10.

ORF	Start	Stop	Strand	Annotation	Database	Best-matched evidence or organism	Accession Num.	%.idt	*E*-value
1	4	291	-	-	NR	-	-	-	-
2	419	628	-	terminase	NR	*Vibrio* phage VapS30	NRA77918.1	54.39	8.37E-16
3	648	1052	-	terminase small subunit	Pfam	*Vibrio* phage VapS30	YEF02900.1	97.02	4.40E-08
4	1421	1789	-	-	-	-	-	-	-
5	1829	2035	-	hypothetical protein	NR	*Vibrio* phage VapS30	YEF02885.1	95.59	1.05E-40
6	2170	2421	-	hypothetical protein	NR	bacterium	MEO2122443.1	61.29	7.29E-18
7	2418	2579	-	hypothetical protein	NR	*Vibrio* phage VapS30	YEF02880.1	94.34	5.48E-28
8	2576	2854	-	hypothetical protein	NR	*Vibrio* phage 11E33.1	QZI86489.1	48.15	3.86E-06
9	2857	3339	-	TreK	Pfam	*Aeromonas* phage PhAER-4	XKC23524.1	50	1.90E-18
10	3717	4151	-	HNH endonuclease	Pfam	Vibrio phage VapS30	YEF02877.1	96.43	1.10E-13
11	4144	4389	-	hypothetical protein	NR	Vibrio phage VapS30	YEF02876.1	87.21	3.18E-43
12	4541	4681	-	-	-	-	-	-	-
13	4771	5706	+	hypothetical protein	NR	Vibrio phage Artemius	UUW39244.1	96.10	0
14	5703	6119	+	putative polynucleotide kinase	NR	*Vibrio* phage 142E35-1	CAH9015111.1	46.38	1.00E-34
15	6138	6617	+	hypothetical protein	NR	*Vibrio* phage VapS30	YEF02919.1	60.12	6.09E-50
16	6601	7155	+	hypothetical protein	NR	*Vibrio* phage Artemius	UUW39241.1	94.05	4.00E-120
17	7152	7448	+	hypothetical protein	NR	*Vibrio* phage Artemius	UUW39240.1	98.98	4.61E-65
18	7496	7909	+	hypothetical protein	NR	Candidatus Bathyarchaeota archaeon	MGD2065586.1	45.93	3.33E-34
19	7920	8207	+	hypothetical protein	NR	*Vibrio* phage Artemius	UUW39239.1	92.63	5.52E-57

20	8197	8892	+	hypothetical protein	NR	*Vibrio* phage VapS30	YEF02915.1	48.78	4.08E-56
21	8894	9655	+	hypothetical protein	NR	*Vibrio* phage Artemius	UUW39237.1	84.83	7.12E-158
22	9658	9978	+	Restriction alleviation protein Lar	Pfam	*Vibrio* phage VapS30	YEF02913.1	60	6.40E-11
23	9983	10306	+	hypothetical protein	NR	*Vibrio* phage VapS30	YEF02912.1	90.65	6.07E-65
24	10395	10685	+	hypothetical protein	NR	*Vibrio* phage 489E54-1	CAH9015623.1	46.24	1.00E-17
25	10672	10869	+	TM helix-containing protein	NR	*Vibrio* phage Artemius	UUW39234.1	98.46	1.00E-37
26	11038	11331	-	TM helix-containing protein	NR	*Vibrio* phage Artemius	UUW39232.1	96.88	2.50E-60
27	11336	11512	-	TM helix-containing protein	NR	*Vibrio* phage 393E50-1	CAH9016001.1	65.45	2.03E-17
28	11512	11850	-	endolysin	NR	*Vibrio* phage pYD21-A	YP_007673974.1	79.46	5.61E-62
29	11837	12043	-	TM helix-containing protein	TrEMBL	ECO:0000256|Google:ProtNLM	A0A2I7S7R1	50.00	7.58E-07
30	12040	12384	-	DUF4326 domain-containing protein	Pfam	*Vibrio* phage Artemius	UUW39229.1	100	1.10E-13
31	12381	14174	-	helicase	Pfam	*Vibrio* phage VapS30	YEF02870.1	98.16	1.70E-17
32	14171	14434	-	hypothetical protein	NR	*Vibrio* phage Artemius	UUW39227.1	91.86	6.02E-51
33	14427	14621	-	Coil-containing protein	TrEMBL	ECO:0000256|Google:ProtNLM	A0A2I7RL57	50.82	2.07E-09
34	14618	14998	-	ATPase	NR	*Vibrio* phage VapS30	YEF02868.1	91.87	4.51E-77
35	15160	16101	+	Coil-containing protein	TrEMBL	ECO:0000256|Google:ProtNLM	A0A2I7S7K4	33.76	6.75E-37
36	16107	17243	+	PD XK nuclease	Pfam	*Vibrio* phage VapS30	YEF02909.1	98.68	8.00E-07
37	17246	17467	+	hypothetical protein	NR	*Vibrio* phage VapS30	YEF02908.1	46.15	1.73E-09
38	17467	18624	+	ssDNA-binding protein	Pfam	*Vibrio* phage Artemius	UUW39223.1	96.36	8.50E-13
39	18630	19064	+	hypothetical protein	NR	*Vibrio* phage VapS30	YEF02906.1	93.42	3.28E-45
40	19064	19309	+	hypothetical protein	NR	*Vibrio* phage Artemius	UUW39221.1	100	2.24E-51

41	19319	21811	+	DNA primase	Pfam	*Vibrio* phage Artemius	UUW39220.1	99.28	4.00E-09
42	21907	22890	+	hypothetical protein	NR	*Vibrio* phage VapS30	YEF02903.1	68.31	2.23E-123
43	22951	25191	+	DNA polymerase	NR	*Vibrio* phage Artemius	UUW39218.1	98.12	0
44	25217	27538	-	tail tubular protein	NR	*Vibrio* phage Artemius	UUW39217.1	75.37	6.49E-82
45	27535	30327	-	TM helix-containing protein	NR	*Vibrio* phage Artemius	UUW39216.1	90.11	0
46	30315	30656	-	hypothetical protein	NR	*Vibrio* phage Artemius	UUW39215.1	97.35	3.00E-76
47	30653	31168	-	hypothetical protein	NR	*Vibrio* phage Artemius	UUW39214.1	100	9.75E-121
48	31165	31662	-	hypothetical protein	NR	*Vibrio* phage VapS30	YEF02862.1	98.79	3.40E-118
49	31662	34496	-	tail length tape measure protein	NR	*Vibrio* phage VapS30	YEF02861.1	96.72	0
50	34493	35014	-	neck protein	NR	*Vibrio* phage Artemius	UUW39211.1	95.95	7.80E-89
51	35241	35642	-	hypothetical protein	NR	*Pseudomonadota* bacterium	MHO4206742.1	99.25	2.48E-91
52	35644	36342	-	tail tube protein	NR	*Vibrio phage Artemius*	UUW39208.1	96.55	2.31E-126
53	36348	36713	-	hypothetical protein	NR	*Pseudomonadota* bacterium	MHO4206744.1	96.69	1.19E-79
54	36714	37526	-	major tail protein	TrEMBL	ECO:0000256|Google:ProtNLM	A0A2I7RIJ2	41.71	2.00E-22
55	37519	37863	-	stopper protein	NR	*Vibrio* phage Artemius	UUW39205.1	97.37	1.31E-75
56	37853	38374	-	hypothetical protein	NR	*Vibrio* phage VapS30	YEF02893.1	98.27	9.91E-123
57	38461	39846	-	phage major capsid protein	Pfam	*Colwellia* sp.	MCP4984700.1	58.98	2.50E-32
58	39933	40586	-	prohead serine protease	Pfam	*Vibrio* phage 2.044.O._10N.261.51.B8	AUS01870.1	73.61	1.10E-21
59	40555	41835	-	portal protein	Pfam	*Vibrio* phage 489E54-1	CAH9015030.1	69.75	3.90E-46
60	41832	43793	-	terminase	Pfam	*Vibrio* phage VapS30	YEF02889.1	92.65	0
61	43783	44025	-	hypothetical protein	NR	*Vibrio* phage Artemius	UUW39199.1	96.25	1.98E-50

Table S3. Homologs of phage vB_ValS-L9 identified by BLASTn searches against the NCBI nucleotide database (performed on October 13, 2026).

No.	Scientific Name	Query Coverage (%)	E-value	Percent identity (%)	GenBank accession ID
1	*Vibrio* phage VapS30	72	0	95.11	PV528705.1
2	*Vibrio* phage Artemius	74	0	95.81	ON366409.1
3	*Vibrio* phage PVA1	4	0	92.03	NC_023605.1
4	*Vibrio* phage vB_VaS_L1	4	0	90.22	OP375635.1
5	*Vibrio* phage 1.072.O._10N.286.48.A12	6	1E-166	73.64	MG592449.1
6	*Vibrio* phage 1.004.O._10N.261.54.A2	6	1E-166	73.64	MG592391.1
7	*Vibrio* phage 1.056.O._10N.261.48.C11	6	1E-166	73.64	MG592436.1
8	*Vibrio* phage 1.068.O._10N.261.51.F8	6	1E-166	73.64	MG592445.1
9	*Vibrio* phage 1.066.O._10N.286.46.E8	6	1E-166	73.64	MG592443.1
10	*Vibrio* phage 1.037.O._10N.261.52.F7	6	1E-166	73.64	MG592421.1
11	*Vibrio* phage 2.044.O._10N.261.51.B8	4	8E-163	73.73	MG592661.1
12	*Vibrio* phage 489E54-1	5	3E-132	75.16	OX241561.1
13	*Vibrio* phage 393E50-1	3	2E-78	71.97	OX241569.1
14	*Vibrio* phage 159E43-1b	1	3E-33	77.15	OZ196825.1
15	*Vibrio* phage 159E36-2b	1	3E-33	77.15	OZ196892.1
16	*Vibrio* phage 159E36-1	1	3E-33	77.15	OZ196842.1
17	*Vibrio* phage 159E36-2a	1	3E-33	77.15	OZ196890.1

Table S4. Comparison of phage vB_ValS-L9 ORFs with homologs in its closely related phages.

**vB_ValS-L9**		**VapS30**			**Artemius**			**1.037.O._10N.261.52.F7**			**159E36-1**		
**ORF**	**Annotation**	**ORF**	**Annotation**	**Identity**	**ORF**	**Annotation**	**Identity**	**ORF**	**Annotation**	**Identity**	**ORF**	**Annotation**	**Identity**
1	-	-	-	-	-	-	-						
2	terminase	-	-	-	-	-	-						
3	terminase small subunit	50	hypothetical protein	97.02	2	terminase small subunit	96.99	1	putative terminase small subunit	57.14	71	hypothetical protein	44.44
4	-	-	-	-	-	-	-						
5	hypothetical protein	49	hypothetical protein	95.59	57	hypothetical protein	92.65						
6	hypothetical protein	-	-	-	-	-	-						
7	hypothetical protein	43	hypothetical protein	94.34	54	DNA-binding domain protein	90.57	48	DNA-binding domain protein	60.38	66	hypothetical protein	58.49
8	hypothetical protein	-	-	-	-	-	-						
9	TreK	-	-	-	-	-	-						
10	HNH endonuclease	40	NH endonuclease	96.43	51	HNH endonuclease	92.14	45	HNH nuclease	61.11			
11	hypothetical protein	39	hypothetical protein	87.29	50	hypothetical protein	82.35	44	hypothetical protein	40.00			
12	-	-	-	-	-	-	-						
13	hypothetical protein	37	hypothetical protein	62.99	49	hypothetical protein	96.10	42	hypothetical protein	29.05	57	hypothetical protein	28.57

14	putative polynucleotide kinase	36	hypothetical protein	82.61	48	hypothetical protein	80.14				23	polynucleotide kinase	47.10
15	hypothetical protein	35	hypothetical protein	60.12	47	hypothetical protein	52.99						
16	hypothetical protein	34	hypothetical protein	80.56	46	hypothetical protein	94.01				53	hypothetical protein	28.00
17	hypothetical protein	-	-	-	45	hypothetical protein	98.98						
18	hypothetical protein	-	-	-	-	-	-						
19	hypothetical protein	33	hypothetical protein	89.47	44	hypothetical protein	92.63						
20	hypothetical protein	31	hypothetical protein	48.78	43	hypothetical protein	58.77						
21	hypothetical protein	30	Mazg nucleotide pyrophosphohydrolase	78.30	42	hypothetical protein	84.83						
22	Restriction alleviation protein Lar	29	hypothetical protein	60.00	-	-	-						
23	hypothetical protein	28	hypothetical protein	90.65	40	hypothetical protein	98.81	38	hypothetical protein	41.43			
24	hypothetical protein	-	-	-	-	-	-						

25	TM helix-containing protein	27	hypothetical protein	98.46	39	TM helix-containing protein	98.46	37	TM helix-containing protein	48.98			
26	TM helix-containing protein	26	hypothetical protein	94.79	37	TM helix-containing protein	96.88	36	hypothetical protein	50.60			
27	TM helix-containing protein	25	hypothetical protein	52.63	36	TM helix-containing protein	56.90	35	TM helix-containing protein	60.71			
28	endolysin	-	-	-	-	-	-						
29	TM helix-containing protein	22	hypothetical protein	90.91	-	-	-	33	hypothetical protein	51.79			
30	DUF4326 domain-containing protein	-	-	-	34	hypothetical protein	100.00						
31	helicase	21	type III restriction enzyme	98.16	33	helicase	92.04	32	helicase superfamily 1/2 ATP-binding domain protein	63.33	36	DNA helicase	41.63
32	hypothetical protein	-	-	-	32	hypothetical protein	91.8						
33	Coil-containing protein	-	-	-	-	-	-						

34	ATPase	19	ATPase	91.87	-	-	-						
35	Coil-containing protein	17	hypothetical protein	97.13	30	hypothetical protein	95.21	28	protein of unknown function DUF2815	40.39	33	hypothetical protein	26.83
36	PD XK nuclease	16	DUF2800 protein	98.68	29	PD XK nuclease	97.88	29	protein of unknown function DUF2800	60.69	31	exonuclease	43.12
37	hypothetical protein	15	hypothetical protein	46.15	-	-	-						
38	ssDNA-binding protein	14	DUF2815 protein	92.21	28	hypothetical protein	96.36	28	protein of unknown function DUF2815	53.18	30	Gp2.5-like ssDNA-binding protein and ssDNA-annealing protein	34.65
39	hypothetical protein	13	hypothetical protein	93.42	27	hypothetical protein	96.53	27	hypothetical protein	74.82	29	hypothetical protein	42.07
40	hypothetical protein	12	hypothetical protein	100.00	26	hypothetical protein	100.00	26	hypothetical protein	29.33			
41	DNA primase	11	hydrolase	99.28	25	DNA primase	99.28	25	DNA primase	58.12	27	DNA polymerase/ primase	46.81
42	hypothetical protein	10	hypothetical protein	68.31	24	hypothetical protein	91.03	24	BRCT domain protein	35.05			

43	DNA polymerase	9	nucleic acid-binding protein	98.93	23	DNA polymerase	98.79	23	hypothetical protein	56.71	21	DNA polymerase	48.18
44	tail tubular protein	7	tail fiber protein	75.76	22	tail tubular protein	75.37						
45	TM helix-containin g protein	6	cellulase	99.57	21	TM helix-containin g protein	90.75				19	tail protein	35.29
46	hypothetical protein	-	-	-	20	hypothetical protein	97.35				18	hypothetical protein	32.91
47	hypothetical protein	5	hypothetical protein	100.00	19	hypothetical protein	100.00				17	hypothetical protein	27.97
48	hypothetical protein	4	hypothetical protein	98.79	18	hypothetical protein	98.18				16	hypothetical protein	29.33
49	tail length tape measure protein	3	tail length tape measure protein	96.72	17	tail length tape measure protein	94.33	16	TM helix-containi ng protein	36.35	15	tail length tape measure protein	46.08
50	neck protein	2	hypothetical protein	100.00	16	neck protein	95.95	15	neck protein	68.02	14	hypothetical protein	30.46
51	hypothetical protein	1	hypothetical protein	100.00	14	hypothetical protein	96.99						
52	tail tube protein	62	glycosyl hydrolase	98.71	13	tail tube protein	96.55	13	hypothetical protein	40.77	11	major tail protein	43.84
53	hypothetical protein	61	hypothetical protein	84.30	12	hypothetical protein	94.22	12	tail-completio n protein	35.11	10	hypothetical protein	27.19

54	major tail protein	-	-	-	11	hypothetical protein	95.93					
55	stopper protein	60	hypothetical protein	96.49	10	Stopper protein	97.37	10	head-tail adaptor	41.67	9	head-closure Hc1	29.73
56	hypothetical protein	59	hypothetical protein	98.27	9	hypothetical protein	98.27	9	head-tail connector protein	32.95	7	head-tail adaptor	28.07
57	phage major capsid protein	57	capsid family protein	52.41	8	major capsid protein	52.63	8	coil-containin g protein	52.02	5	major head protein	51.41
58	prohead serine protease	56	N-acetylmura moyl-L-alanin e amidase	55.45	7	hypothetical protein	55.45	7	prohead protease	73.61	4	head maturation protease	61.00
59	portal protein	55	portal protein	68.70	6	portal protein	68.46	6	gene transfer agent portal protein	69.48	3	portal protein	43.00
60	terminase	54	terminase	92.65	5	terminase large subunit	73.17	5	terminase	69.07	1	terminase large subunit	50.08
61	hypothetical protein	53	hypothetical protein	98.67	4	hypothetical protein	96.25	3	hypothetical protein	64.00			

References

1. Lu, L.; Ni, L.; Ai, C.; Zhang, D.; Zheng, P.; Li, X.; Liu, X.; Dang, H. Seasonal dynamics of microbial communities link to summer-autumn aquaculture disease outbreaks in Sanggou Bay. *Front. Mar. Sci.* **2025**, *12*, 1581190. https://doi.org/10.3389/fmars.2025.1581190.
2. Li, X.; Liang, Y.; Wang, Z.; Yao, Y.; Chen, X.; Shao, A.; Lu, L.; Dang, H. Isolation and characterization of a novel *Vibrio natriegens*-infecting phage and its potential therapeutic application in abalone aquaculture. *Biology* **2022**, *11*, 1670. https://doi.org/10.3390/biology11111670.
3. Chang, L.; Wei, Z.; Lu, L.; Liu, L.; Yao, Y.; Ding, W.; Ni, T.; Chen, X.; Wang, H.; Bao, Z.; Hu, X. *Vibrio alginolyticus* AbY-1904: A neotype pathogen and its pathogenic characterization in *Haliotis discus hannai*. *Aquaculture* **2025**, *602*, 742322. https://doi.org/10.1016/j.aquaculture.2025.742322.
